# Supplementary material for: ProSPective evaluation of the dIagnostic accuracy of siNe spiN non-contrast flatdEtectoR CT (FDCT) for the detection of intracranial hemorrhage in stroke patients - Protocol of a non-inferiority comparison to multi detector CT
Source: PLoS One. 2025 Aug 28;20(8):e0330608. doi: 10.1371/journal.pone.0330608 (PMC12393753; doi:10.1371/journal.pone.0330608)
Supplement: S1 File — This file includes an overview of sites, investigators and the supplementary tables: S1 table. Overview of possible outcomes for the readings; S2 table. Trial registration: data set; S3 table. SPIRIT Checklist for Trials. (SPINNERS_SupplementaryAppendix.DOCX) [file pone.0330608.s001.docx]

**Supplementary Appendix**

**ProSPective evaluation of the dIagnostic accuracy of siNe spiN non-contrast flat-dEtectoR CT (FDCT) for the detection of intracranial hemorrhage in Stroke patients**

Supplement to: Psychogios M, Brehm A, Goyal N et al. ProSPective evaluation of the dIagnostic accuracy of siNe spiN non-contrast flatdEtectoR CT (FDCT) for the detection of intracranial hemorrhage in stroke patients - Protocol of a non-inferiority comparison to multi detector CT

This appendix has been provided by the authors to give readers additional information about the work.

**Contents**

[I. Listing of participating sites and investigators in SPINNERS 3](#_Toc191373031)

[1. Participating active centers, site investigators and site main investigator(s) 3](#_Toc191373032)

[2. SPINNERS executive committee 5](#_Toc191373033)

[3. Data safety and monitoring committee 5](#_Toc191373034)

[4. Sponsor Team 5](#_Toc191373035)

[II. Supplementary tables 6](#_Toc191373036)

[Table S1. Overview of possible outcomes for the readings 6](#_Toc191373037)

[Table S2. Trial registration: data set 6](#_Toc191373038)

[Table S3. SPIRIT Checklist for *Trials* 8](#_Toc191373039)

# Listing of participating sites and investigators in SPINNERS

## Participating active centers, site investigators and site main investigator(s)

| Site Name | Site investigators | Site main investigators |  |
| --- | --- | --- | --- |
| **SWITZERLAND** | | | |
| University Hospital Basel | Jehuda Soleman  Kristine Ann Blackham  Victor Schulze-Zachau  Anh Nguyen  Nikolaos Ntoulias | Marios Psychogios  Ioannis Tsogkas |  |
| University Hospital of Bern, Inselspital | Tomas Dobrocky  Eike Piechowiak  Sara Pilgram-Pastor  David Seiffge  Roman Rohner | Johannes Kaesmacher |  |
| Luzerner Kantonsspital, Luzern | Manuel Bolognese  Lehel Lakatos  Christian Kamm  Stephan Bohlhalter  Marie Guillaume | Alexander von Hessling  Grzegorz Marek Karwacki |  |

| Site Name | Site investigators | Site main investigators |  |
| --- | --- | --- | --- |
| **United States of America** | | | |
| Semmes Murphey Clinic and University of Tennessee Health Sciences Center, Memphis | Violiza Inoa  Christopher Nickele  Nicklaus Khan  Daniel Hoit  Lucas Elijovich | Adam S. Arthur  Nitin Goyal |  |
| Hospital of the University of Pennsylvania, Philadelphia | Visish Srinivasan  Josh Catapano  Bryan Pukenas  Sandeep Kandregula  Redi Rahmani | Jan-Karl Burkhardt |  |
| Advocate Medical Group, Park Ridge | Marion Oliver  Krishna Joshi  Josh Billingsley  Kiffon Keigher  Joao Victor Sanders | Demetrius K Lopes |  |
| Endeavor Health, Chicago | William Ares | Shakeel A. Chowdhry |  |
| University of Virginia | Pedro Norat | Ryan T Kellog |  |
| Mt Sinai Hospital Systems, New York |  | Christopher Kellner |  |
| NYU Langone Health, New York | Maksim Shapiro  Vera Sharashidze  Erez Nossek  Svetlana Kvint  Rogelio Esparza | Eytan Raz |  |
| RIA Neurovascular, Englewood |  | Donald Frei |  |

| Site Name | Site investigators | Site main investigators |  |
| --- | --- | --- | --- |
| **Helsinki** | | | |
| Helsinki University Hospital | Mikko Sillanpää  Tatu Kokkonen  Riikka Lauha  Liisa Tomppo  Henrietta Törmänen | Daniel Strbian  Laura Mannismäki |  |

| Site Name | Site investigators | Site main investigators |  |
| --- | --- | --- | --- |
| **Spain** | | | |
| Hospital Vall d’Hebron Barcelona | Manuel Requena  Francesco Diana  David Hernandez  Marta De Dios | Alejandro Tomasello |  |

| Site Name | Site investigators | Site main investigators |  |
| --- | --- | --- | --- |
| **France** | | | |
| CHRU Tours, Tours | Kevin Janot  Marco Pasi  Fouzi Bala  Nouroudine (Adeniran) Bankole  Abder el Houfia | Grégoire Boulouis |  |

## SPINNERS executive committee

| Marios Psychogios | Diagnostic and Interventional Neuroradiology, University Hospital Basel, Switzerland |
| --- | --- |
| Adam Arthur | Semmes Murphey Clinic and University of Tennessee Health Sciences Center, Memphis |
| Nikki Rommers | Department Clinical Research, University Hospital Basel, Switzerland |

## Data safety and monitoring committee

| Georgios Tsivgoulis (Chair) | Second Department of Neurology**,** National & Kapodistrian University of Athens, Greece |
| --- | --- |
| Antonia Zapf (Statistician) | Institute of Medical Biometry and Epidemiology**,** University Hospital Hamburg Eppendorf, Hamburg, Germany |
| Robert M. Starke (Member) | Department of Neurological Surgery, Miller School of Medicine, University of Miami, Florida, USA |

## Sponsor Team

| Luzia Balmer | Project Manager, Diagnostic and Interventional Neuroradiology, University Hospital Basel, Switzerland |
| --- | --- |
| Vera Aebischer | Imaging support/ PhD Fellow, Diagnostic and Interventional Neuroradiology, University Hospital Basel, Switzerland |
| Aikaterini Anastasiou | Imaging support/ PhD Fellow, Diagnostic and Interventional Neuroradiology, University Hospital Basel, Switzerland |
| Tamara Zeschky | Data Manager, Department Clinical Research, University Hospital Basel, Switzerland |
| Florian Peters | Clinical Monitor, Department Clinical Research, University Hospital Basel, Switzerland |
| Nastassia Trachsel | Clinical Monitor, Department Clinical Research, University Hospital Basel, Switzerland |

# Supplementary tables

## S1 table. Overview of possible outcomes for the readings

| Ground truth | Core-Lab 1 | Core-Lab 2 | Final Result |
| --- | --- | --- | --- |
| MDCT | FDCT | FDCT | FDCT |
| (+) | (+) | NA | True (+) |
| (-) | (-) | NA | True (-) |
| (+) | (-) | NA | False (-) |
| (-) | (+) | (-) | False (+) |
| (-) | (+) | (+) | Hemorrhage occurred between scans |

FDCT Flat-Detector CT, MDCT Multi-Detector CT

## S2 table. Trial registration: data set

| **Data category** | **Information** |
| --- | --- |
| Primary registry and trial identifying number | ClinicalTrials.gov Identifier: NCT05458908 |
| Date of registration in primary registry | 13.July 2022 |
| Secondary identifying numbers | NA |
| Source(s) of monetary or material support | Siemens Healthineers AG |
| Primary sponsor | University Hospital Basel, Basel, Switzerland |
| Secondary sponsor(s) | NA |
| Contact for public queries | [Marios.psychogios@usb.ch](mailto:Marios.psychogios@usb.ch); alex.brehm@usb.ch |
| Contact for scientific queries | [Marios.psychogios@usb.ch](mailto:Marios.psychogios@usb.ch); alex.brehm@usb.ch |
| Public title | ProSPective Evaluation of Non-contrast sINe spiN Flat-dEtectoR CT for the Detection of Intracranial hemorrhageS (SPINNERS) |
| Scientific title | ProSPective evaluation of the dIagnostic accuracy of siNe spiN non-contrast flat-dEtectoR CT (FDCT) for the detection of intracranial hemorrhage in Stroke patients - an open labelled, multicenter, non-inferiority comparison of FDCT to multi detector CT (MDCT) with blinded assessment of outcome events |
| Countries of recruitment | Switzerland, United States of America, Spain, Finland, France |
| Health condition(s) or problem(s) studied | Stroke |
| Intervention(s) | Diagnostic Test: Non-contrast cranial MDCT head scan |
|  | Diagnostic Test: Non-contrast syngo DynaCT Sine Spin head scan and application software |
| Key inclusion and exclusion criteria | Ages eligible for study: ≥18 years  Sexes eligible for study: both  Accepts healthy volunteers: no |
|  | Key Inclusion criteria: atients with symptoms suggestive of ischemic stroke (NIHSS ≥ 7) or suggestive of haemorrhagic stroke with a cranial non-contrast MDCT and a feasible non-contrast syngo DynaCT Sine Spin within 4 hours; Patient presenting within 24 hours of last seen well; Patients presenting directly to the treating hospital (i.e. mothership patients) OR transfer patients with the indication for repeated imaging according to the standard operation procedures of the treating hospital |
|  | Key Exclusion criteria: Severe metal artifacts on initial MDCT imaging; Planned invasive interventions between MDCT and FDCT scan |
| Study type | Diagnostic |
|  | Single group assignment |
|  | Cross-sectional non-inferiority investigation with prospective open label data collection and blinded endpoint assessment |
| Date of first enrolment | 25. October 2022 |
| Target sample size | 252 |
| Recruitment status | Recruiting |
| Primary outcome(s) | The primary outcome is the occurrence of intracranial hemorrhages (yes vs no) as assessed by a blinded core-lab. The primary outcome will be used to calculate the sensitivity and specificity of non-contrast syngo DynaCT Sine Spin imaging for the detection of intracranial hemorrhages. |
| Key secondary outcomes | NA |

## S3 table. SPIRIT Checklist for *Trials*

Complete this checklist by entering the page and line numbers where each of the items listed below can be found in your manuscript.

Your manuscript may not currently address all the items on the checklist. Please modify your text to include the missing information. If you are certain that an item does not apply, please state "n/a" and provide a short explanation. **Leaving an item blank or stating “n/a” without an explanation will lead to your manuscript being returned before review.**

Upload your completed checklist as an additional file when you submit to *Trials*. You must reference this additional file in the main text of your protocol submission. The completed SPIRIT figure must be included within the main body of the protocol text and can be downloaded here: <http://www.spirit-statement.org/schedule-of-enrolment-interventions-and-assessments/>

In your methods section, please state that you used the SPIRIT reporting guidelines, and cite them as:

Chan A-W, Tetzlaff JM, Gøtzsche PC, Altman DG, Mann H, Berlin J, Dickersin K, Hróbjartsson A, Schulz KF, Parulekar WR, Krleža-Jerić K, Laupacis A, Moher D. SPIRIT 2013 Explanation and Elaboration: Guidance for protocols of clinical trials. BMJ. 2013;346:e7586

|  |  | **Reporting Item** | **Page and Line Number** | **Reason if not applicable** |
| --- | --- | --- | --- | --- |
| **Administrative information** | | | | |
| Title | [#1](https://www.goodreports.org/reporting-checklists/spirit/info/#1) | Descriptive title identifying the study design, population, interventions, and, if applicable, trial acronym | Page 1 line 1 – 4 |  |
| Trial registration | [#2a](https://www.goodreports.org/reporting-checklists/spirit/info/#2a) | Trial identifier and registry name. If not yet registered, name of intended registry | Page 3 line 75 |  |
| Trial registration: data set | [#2b](https://www.goodreports.org/reporting-checklists/spirit/info/#2b) | All items from the World Health Organization Trial Registration Data Set | Supplement table S2 |  |
| Protocol version | [#3](https://www.goodreports.org/reporting-checklists/spirit/info/#3) | Date and version identifier | Page 5, line 103 |  |
| Funding | [#4](https://www.goodreports.org/reporting-checklists/spirit/info/#4) | Sources and types of financial, material, and other support | Page 9 line 227-228 |  |
| Roles and responsibilities: contributorship | [#5a](https://www.goodreports.org/reporting-checklists/spirit/info/#5a) | Names, affiliations, and roles of protocol contributors | Page 9 line 225- 236 |  |
| Roles and responsibilities: sponsor contact information | [#5b](https://www.goodreports.org/reporting-checklists/spirit/info/#5b) | Name and contact information for the trial sponsor | Page 9 line 225 - 236 |  |
| Roles and responsibilities: sponsor and funder | [#5c](https://www.goodreports.org/reporting-checklists/spirit/info/#5c) | Role of study sponsor and funders, if any, in study design; collection, management, analysis, and interpretation of data; writing of the report; and the decision to submit the report for publication, including whether they will have ultimate authority over any of these activities | Page 9 line 220 – 236 |  |
| Roles and responsibilities: committees | [#5d](https://www.goodreports.org/reporting-checklists/spirit/info/#5d) | Composition, roles, and responsibilities of the coordinating centre, steering committee, endpoint adjudication committee, data management team, and other individuals or groups overseeing the trial, if applicable (see Item 21a for data monitoring committee) | Page 6 line 152 – page 7 line 179  Page 9 line 214 – 217 |  |
| **Introduction** |  |  |  |  |
| Background and rationale | [#6a](https://www.goodreports.org/reporting-checklists/spirit/info/#6a) | Description of research question and justification for undertaking the trial, including summary of relevant studies (published and unpublished) examining benefits and harms for each intervention | Page 4 line 83– 98 |  |
| Background and rationale: choice of comparators | [#6b](https://www.goodreports.org/reporting-checklists/spirit/info/#6b) | Explanation for choice of comparators | Page 7 line 160 -167 |  |
| Objectives | [#7](https://www.goodreports.org/reporting-checklists/spirit/info/#7) | Specific objectives or hypotheses | Page 5 line 107 - 109 |  |
| Trial design | [#8](https://www.goodreports.org/reporting-checklists/spirit/info/#8) | Description of trial design including type of trial (eg, parallel group, crossover, factorial, single group), allocation ratio, and framework (eg, superiority, equivalence, non-inferiority, exploratory) | Page 5 line 102 - 111 |  |
| **Methods: Participants, interventions, and outcomes** | | | | |
| Study setting | [#9](https://www.goodreports.org/reporting-checklists/spirit/info/#9) | Description of study settings (eg, community clinic, academic hospital) and list of countries where data will be collected. Reference to where list of study sites can be obtained | Page 6 line 136 - 140 |  |
| Eligibility criteria | [#10](https://www.goodreports.org/reporting-checklists/spirit/info/#10) | Inclusion and exclusion criteria for participants. If applicable, eligibility criteria for study centres and individuals who will perform the interventions (eg, surgeons, psychotherapists) | Table 1 |  |
| Interventions: description | [#11a](https://www.goodreports.org/reporting-checklists/spirit/info/#11a) | Interventions for each group with sufficient detail to allow replication, including how and when they will be administered | Page 6 line 152 – page 7 line 157 |  |
| Interventions: modifications | [#11b](https://www.goodreports.org/reporting-checklists/spirit/info/#11b) | Criteria for discontinuing or modifying allocated interventions for a given trial participant (eg, drug dose change in response to harms, participant request, or improving / worsening disease) |  | Not applicable as it is a single intervention and no modification is allowed |
| Interventions: adherance | [#11c](https://www.goodreports.org/reporting-checklists/spirit/info/#11c) | Strategies to improve adherence to intervention protocols, and any procedures for monitoring adherence (eg, drug tablet return; laboratory tests) |  | Not applicable as the intervention happens at only one time point no follow-ups |
| Interventions: concomitant care | [#11d](https://www.goodreports.org/reporting-checklists/spirit/info/#11d) | Relevant concomitant care and interventions that are permitted or prohibited during the trial |  | Not applicable as all concomitant care is allowed |
| Outcomes | [#12](https://www.goodreports.org/reporting-checklists/spirit/info/#12) | Primary, secondary, and other outcomes, including the specific measurement variable (eg, systolic blood pressure), analysis metric (eg, change from baseline, final value, time to event), method of aggregation (eg, median, proportion), and time point for each outcome. Explanation of the clinical relevance of chosen efficacy and harm outcomes is strongly recommended | Page 7 line 158 – page 8 line 185 |  |
| Participant timeline | [#13](https://www.goodreports.org/reporting-checklists/spirit/info/#13) | Time schedule of enrolment, interventions (including any run-ins and washouts), assessments, and visits for participants. A schematic diagram is highly recommended (see Figure) | Figure 1 and figure 2 |  |
| Sample size | [#14](https://www.goodreports.org/reporting-checklists/spirit/info/#14) | Estimated number of participants needed to achieve study objectives and how it was determined, including clinical and statistical assumptions supporting any sample size calculations | Page 8 line 191 - 209 |  |
| Recruitment | [#15](https://www.goodreports.org/reporting-checklists/spirit/info/#15) | Strategies for achieving adequate participant enrolment to reach target sample size |  | Not applicable as enrollment happens in the acute phase of the treatment of the patient |
| **Methods: Assignment of interventions (for controlled trials)** | | | | |
| Allocation: sequence generation | [#16a](https://www.goodreports.org/reporting-checklists/spirit/info/#16a) | Method of generating the allocation sequence (eg, computer-generated random numbers), and list of any factors for stratification. To reduce predictability of a random sequence, details of any planned restriction (eg, blocking) should be provided in a separate document that is unavailable to those who enrol participants or assign interventions |  | Not applicable as no RCT |
| Allocation concealment mechanism | [#16b](https://www.goodreports.org/reporting-checklists/spirit/info/#16b) | Mechanism of implementing the allocation sequence (eg, central telephone; sequentially numbered, opaque, sealed envelopes), describing any steps to conceal the sequence until interventions are assigned |  | Not applicable as no RCT |
| Allocation: implementation | [#16c](https://www.goodreports.org/reporting-checklists/spirit/info/#16c) | Who will generate the allocation sequence, who will enrol participants, and who will assign participants to interventions |  | Not applicable as no RCT |
| Blinding (masking) | [#17a](https://www.goodreports.org/reporting-checklists/spirit/info/#17a) | Who will be blinded after assignment to interventions (eg, trial participants, care providers, outcome assessors, data analysts), and how | Page 6 line 146 - 151 |  |
| Blinding (masking): emergency unblinding | [#17b](https://www.goodreports.org/reporting-checklists/spirit/info/#17b) | If blinded, circumstances under which unblinding is permissible, and procedure for revealing a participant’s allocated intervention during the trial |  | Not applicable as no RCT |
| **Methods: Data collection, management, and analysis** | | | | |
| Data collection plan | [#18a](https://www.goodreports.org/reporting-checklists/spirit/info/#18a) | Plans for assessment and collection of outcome, baseline, and other trial data, including any related processes to promote data quality (eg, duplicate measurements, training of assessors) and a description of study instruments (eg, questionnaires, laboratory tests) along with their reliability and validity, if known. Reference to where data collection forms can be found, if not in the protocol | Page 6 line 132 – 133 Page 7 158 - 185 |  |
| Data collection plan: retention | [#18b](https://www.goodreports.org/reporting-checklists/spirit/info/#18b) | Plans to promote participant retention and complete follow-up, including list of any outcome data to be collected for participants who discontinue or deviate from intervention protocols |  | Not applicable as no follow-ups are done |
| Data management | [#19](https://www.goodreports.org/reporting-checklists/spirit/info/#19) | Plans for data entry, coding, security, and storage, including any related processes to promote data quality (eg, double data entry; range checks for data values). Reference to where details of data management procedures can be found, if not in the protocol | Page 6 line 132 – 133 |  |
| Statistics: outcomes | [#20a](https://www.goodreports.org/reporting-checklists/spirit/info/#20a) | Statistical methods for analysing primary and secondary outcomes. Reference to where other details of the statistical analysis plan can be found, if not in the protocol | Page 8 line 210 – page 9 line 219 |  |
| Statistics: additional analyses | [#20b](https://www.goodreports.org/reporting-checklists/spirit/info/#20b) | Methods for any additional analyses (eg, subgroup and adjusted analyses) |  | Not applicable as no additional analysis are done |
| Statistics: analysis population and missing data | [#20c](https://www.goodreports.org/reporting-checklists/spirit/info/#20c) | Definition of analysis population relating to protocol non-adherence (eg, as randomised analysis), and any statistical methods to handle missing data (eg, multiple imputation) | Page 8 line 210 – page 9 line 219 |  |
| **Methods: Monitoring** | | | | |
| Data monitoring: formal committee | [#21a](https://www.goodreports.org/reporting-checklists/spirit/info/#21a) | Composition of data monitoring committee (DMC); summary of its role and reporting structure; statement of whether it is independent from the sponsor and competing interests; and reference to where further details about its charter can be found, if not in the protocol. Alternatively, an explanation of why a DMC is not needed | Page 9 line 220- 224 |  |
| Data monitoring: interim analysis | [#21b](https://www.goodreports.org/reporting-checklists/spirit/info/#21b) | Description of any interim analyses and stopping guidelines, including who will have access to these interim results and make the final decision to terminate the trial | Page 9 line 220 - 224 |  |
| Harms | [#22](https://www.goodreports.org/reporting-checklists/spirit/info/#22) | Plans for collecting, assessing, reporting, and managing solicited and spontaneously reported adverse events and other unintended effects of trial interventions or trial conduct | Page 9 line 220 - 224 |  |
| Auditing | [#23](https://www.goodreports.org/reporting-checklists/spirit/info/#23) | Frequency and procedures for auditing trial conduct, if any, and whether the process will be independent from investigators and the sponsor |  | Not applicable as no audit is planned |
| **Ethics and dissemination** | | | | |
| Research ethics approval | [#24](https://www.goodreports.org/reporting-checklists/spirit/info/#24) | Plans for seeking research ethics committee / institutional review board (REC / IRB) approval | Protocol v2.1 dated 29.04.2024 page 26 -27 |  |
| Protocol amendments | [#25](https://www.goodreports.org/reporting-checklists/spirit/info/#25) | Plans for communicating important protocol modifications (eg, changes to eligibility criteria, outcomes, analyses) to relevant parties (eg, investigators, REC / IRBs, trial participants, trial registries, journals, regulators) | Protocol v2.1 dated 29.04.2024 page 30 |  |
| Consent or assent | [#26a](https://www.goodreports.org/reporting-checklists/spirit/info/#26a) | Who will obtain informed consent or assent from potential trial participants or authorised surrogates, and how (see Item 32) | Protocol v2.1 dated 29.04.2024 page 28 - 29 |  |
| Consent or assent: ancillary studies | [#26b](https://www.goodreports.org/reporting-checklists/spirit/info/#26b) | Additional consent provisions for collection and use of participant data and biological specimens in ancillary studies, if applicable |  | NA as no additional consent provisions are foreseen |
| Confidentiality | [#27](https://www.goodreports.org/reporting-checklists/spirit/info/#27) | How personal information about potential and enrolled participants will be collected, shared, and maintained in order to protect confidentiality before, during, and after the trial | Protocol v2.1 dated 29.04.2024 page 29 and 67 |  |
| Declaration of interests | [#28](https://www.goodreports.org/reporting-checklists/spirit/info/#28) | Financial and other competing interests for principal investigators for the overall trial and each study site | Protocol v2.1 dated 29.04.2024 page 27 |  |
| Data access | [#29](https://www.goodreports.org/reporting-checklists/spirit/info/#29) | Statement of who will have access to the final trial dataset, and disclosure of contractual agreements that limit such access for investigators | Protocol v2.1 dated 29.04.2024 page 69 |  |
| Ancillary and post trial care | [#30](https://www.goodreports.org/reporting-checklists/spirit/info/#30) | Provisions, if any, for ancillary and post-trial care, and for compensation to those who suffer harm from trial participation |  | NA as no post-trial care is foreseen |
| Dissemination policy: trial results | [#31a](https://www.goodreports.org/reporting-checklists/spirit/info/#31a) | Plans for investigators and sponsor to communicate trial results to participants, healthcare professionals, the public, and other relevant groups (eg, via publication, reporting in results databases, or other data sharing arrangements), including any publication restrictions | Protocol v2.1 dated 29.04.2024 page 69 |  |
| Dissemination policy: authorship | [#31b](https://www.goodreports.org/reporting-checklists/spirit/info/#31b) | Authorship eligibility guidelines and any intended use of professional writers | Protocol v2.1 dated 29.04.2024 page 69 |  |
| Dissemination policy: reproducible research | [#31c](https://www.goodreports.org/reporting-checklists/spirit/info/#31c) | Plans, if any, for granting public access to the full protocol, participant-level dataset, and statistical code | Protocol v2.1 dated 29.04.2024 page 69 |  |
| **Appendices** | | | | |
| Informed consent materials | [#32](https://www.goodreports.org/reporting-checklists/spirit/info/#32) | Model consent form and other related documentation given to participants and authorised surrogates |  | NA |
| Biological specimens | [#33](https://www.goodreports.org/reporting-checklists/spirit/info/#33) | Plans for collection, laboratory evaluation, and storage of biological specimens for genetic or molecular analysis in the current trial and for future use in ancillary studies, if applicable |  | NA |

It is strongly recommended that this checklist be read in conjunction with the SPIRIT 2013 Explanation & Elaboration for important clarification on the items. Amendments to the protocol should be tracked and dated. The SPIRIT checklist is copyrighted by the SPIRIT Group under the Creative Commons “[Attribution-NonCommercial-NoDerivs 3.0 Unported](http://www.creativecommons.org/licenses/by-nc-nd/3.0/)” license. This checklist can be completed online using https://www.goodreports.org/, a tool made by the EQUATOR Network in collaboration with Penelope.ai
