# Supplementary material for: ProSPective evaluation of the dIagnostic accuracy of siNe spiN non-contrast flatdEtectoR CT (FDCT) for the detection of intracranial hemorrhage in stroke patients - Protocol of a non-inferiority comparison to multi detector CT
Source: PLoS One. 2025 Aug 28;20(8):e0330608. doi: 10.1371/journal.pone.0330608 (PMC12393753; doi:10.1371/journal.pone.0330608)
Supplement: S2 File — This file includes the final protocol (V2.1 dated 29.04.2024) of the SPINNERS trial. (DOCX) [file pone.0330608.s002.docx]

**Clinical Investigation Plan**

**ProSPective evaluation of the dIagnostic accuracy of siNe spiN non-contrast flat-dEtectoR CT (FDCT) for the detection of intracranial hemorrhage in Stroke patients**

**-**

**an open labelled, multicenter, non-inferiority comparison of FDCT to multi detector CT (MDCT) with blinded assessment of outcome events**

**SPINNERS Trial**

| Type of investigation | Clinical investigation concerning medical devices (MD). |
| --- | --- |
| Categorization | According to Swiss legislation (Human research act (HRA)^1^ and Art 6 ClinO-MD^2^) the clinical investigation is categorized as a clinical investigation concerning a medical device class A2, since the medical device under investigation is CE-certified, commercially available in Switzerland and it will be used within its intended use. As there will be minor excess radiation^3^ in a subgroup of enrolled subjects, the clinical investigation is rated as A2.^4^  US medical device classification Class 2. Minimal risk to subject (up to 2.5 mSv according to internal measurements and recent publications) |
| Identifier | Clinicaltrials.gov (NCT05458908) |
| Sponsor-Investigator | Prof. Dr. Marios-Nikos Psychogios, M.D. (main contact person)  University Hospital Basel,  Petersgraben 4  CH-4031 Basel,  Switzerland |
| CO-Principal-Investigator / Chair USA | Dr. Adam S. Arthur, M.D., M.P.H. (Chair USA)  Semmes Murphey Clinic and University of Tennessee Health Sciences Center  Memphis TN 38120,  6325 Humphreys Blvd  USA |
| Medical Device | ARTIS icono biplane angiography system, model no.: 11327600, software version VE21 or higher with syngo application software, model no.: 11327667, version VE21D or higher.  UDI-DI ARTIS icono: 04056869063317  UDI-DI syngo application software: 04056869124162  US 510(k) Number:  ARTIS icono K201156  syngo application software K190780 |
| CIP Version and date | Version 2.1 29.04.2024 |

| **Version** | **Autor** | **Reviewed by** | **Change** | **Date** |
| --- | --- | --- | --- | --- |
| 1.0 | Alex Brehm | Marios-Nikos Psychogios, Adam Arthur | New Document | 15.02.2022 |
| 1.1 | Alex Brehm | Marios-Nikos Psychogios, Adam Arthur | DSMC entered, minor changes on the background of the study | 24.03.2022 |
| 1.2 | Alex Brehm | Marios-Nikos Psychogios, Adam Arthur | Corrections according to letter from EKNZ and minor changes | 31.05.2022 |
| 1.3 | Alex Brehm | - | Clinicaltrials.gov identifier added | 25.07.2022 |
| 1.4 | Alex Brehm |  | *Software Version*  *was changed to*  *VE21 or higher* | 22.09.2022 |
| 1.5 | Chad Burleson/Alex Brehm |  | US/French Amendments added /Contact information for Chad Burleson updated | 08.05.2023 |
| 2.0 | Alex Brehm | Marios-Nikos Psychogios, Adam Arthur | Clarification of criteria for enrolment of transfer patient, Change in enrolment process, minor text changes for clarity and uniformity | 28.07.2023 |
| 2.1 | Alex Brehm | Marios-Nikos Psychogios, Adam Arthur | Handling of operator or device failure for obtaining images | 29.04.2024 |

**Signature Page(s)**

| ID number of the investigation: | *Clinicaltrials.gov (XX) and kofam* |
| --- | --- |
| Title: | *ProSPective evaluation of the dIagnostic accuracy of siNe spiN non-contrast flat-dEtectoR CT (FDCT) for the detection of intracranial hemorrhage in Stroke patients - an open labelled, multicenter, non-inferiority comparison of FDCT to multi detector CT (MDCT) with blinded assessment of outcome events (SPINNERS Trial)* |

The Sponsor-Investigator, the US Chair and the Statistician have approved the CIP version 2.1 (29.04.2024) and confirm hereby to conduct the investigation according to the CIP, the current version of the World Medical Association Declaration of Helsinki, the ISO14155 norm, and ICH-GCP as far as applicable, and the local legally applicable requirements.

Sponsor-Investigator: Marios-Nikos Psychogios

| Place/Date |  | Signature |
| --- | --- | --- |

**Statistician: Nikki Rommers, PhD:**

| Place/Date |  | Signature |
| --- | --- | --- |

**US-Chair: Adam Arthur, MD, MPH**

| Place/Date |  | Signature |
| --- | --- | --- |

**Principal Investigator at the local investigational site:**

I have read and understood this CIP version (Version 21, 29.04.2024), and agree to conduct the investigation according to the CIP, the current version of the World Medical Association Declaration of Helsinki, the ISO14155 norm, and ICH-GCP as far as applicable, and the local legally applicable requirements.

| Site: |  |
| --- | --- |
| Principal investigator at the local investigational site: |  |

| Place/Date |  | Signature |
| --- | --- | --- |

**Table of Contents**

[SYNOPSIS 11](#_Toc106024760)

[ABBREVIATIONS 18](#_Toc106024761)

[INVESTIGATION SCHEDULE 20](#_Toc106024762)

[1. INVESTIGATION ADMINISTRATIVE STRUCTURE 21](#_Toc106024763)

[1.1 Sponsor, Sponsor-Investigator 21](#_Toc106024764)

[1.2 CO-PI and US-Chair 21](#_Toc106024765)

[1.3 Principial Investigators (PIs) in Switzerland and abroad 21](#_Toc106024766)

[1.4 Statistician 21](#_Toc106024767)

[1.5 Laboratory 22](#_Toc106024768)

[1.6 Monitoring Institution 22](#_Toc106024769)

[1.7 Data Safety Monitoring Committee (DSMC) 22](#_Toc106024770)

[1.8 Primary Imaging Core Lab (ICL) 23](#_Toc106024771)

[1.9 Secondary Imaging Core Lab (ICL) 23](#_Toc106024772)

[1.10 Trial Management and Administrative Lead 24](#_Toc106024773)

[1.11 Data Management 24](#_Toc106024774)

[2. ETHICAL AND REGULATORY ASPECTS 24](#_Toc106024775)

[2.1 Registration of the investigation 26](#_Toc106024776)

[2.2 Categorization of the investigation 26](#_Toc106024777)

[2.3 Competent Ethics Committee (CEC) 26](#_Toc106024778)

[2.3.1 Reporting duties to the Competent Ethics Committee 27](#_Toc106024779)

[2.4 Competent Authorities (CA) 27](#_Toc106024780)

[2.5 Ethical Conduct of the Trial 27](#_Toc106024781)

[2.6 Declaration of interest 27](#_Toc106024782)

[2.7 Patient Information and Informed Consent 28](#_Toc106024783)

[2.8 Subject privacy and confidentiality 29](#_Toc106024784)

[2.9 Early termination of the Investigation 29](#_Toc106024785)

[2.10 Clinical investigation plan amendments 30](#_Toc106024786)

[2.11 Deviation from the Clinical Investigation Plan 30](#_Toc106024787)

[2.12 Pregnancy 31](#_Toc106024788)

[3. BACKGROUND AND RATIONALE 32](#_Toc106024789)

[3.1 Background and Rationale for the clinical investigation 32](#_Toc106024790)

[3.2 Identification and description of the Investigational Medical Device 33](#_Toc106024791)

[3.3 Preclinical evidence 36](#_Toc106024792)

[3.4 Clinical Evidence to Date 36](#_Toc106024793)

[3.4.1 Systematic review: Search strategy 36](#_Toc106024794)

[3.4.2 Clinical evidence to Date 36](#_Toc106024795)

[3.5 Justification for the design of the clinical investigation 37](#_Toc106024796)

[3.6 Explanation for choice of comparator/ground truth 37](#_Toc106024797)

[3.7 Risk evaluation (Risk-to-Benefits rationale) 38](#_Toc106024798)

[3.8 Justification of the choice of the investigation population 40](#_Toc106024799)

[4. CLINICAL INVESTIGATION OBJECTIVES 41](#_Toc106024800)

[4.1 Overall Objective 41](#_Toc106024801)

[4.2 Primary Objective 41](#_Toc106024802)

[4.3 Secondary Objectives 41](#_Toc106024803)

[4.4 Safety Objectives 41](#_Toc106024804)

[5. CLINICAL INVESTIGATION OUTCOMES 42](#_Toc106024805)

[5.1 Primary Outcome 42](#_Toc106024806)

[5.2 Safety Outcomes 42](#_Toc106024807)

[6. CLINICAL INVESTIGATION DESIGN 43](#_Toc106024808)

[6.1 General clinical investigation design and justification of design 43](#_Toc106024809)

[6.2 Methods for minimizing bias 46](#_Toc106024810)

[6.2.1 Randomisation 46](#_Toc106024811)

[6.2.2 Blinding procedures 46](#_Toc106024812)

[6.2.3 Other methods for minimising bias 47](#_Toc106024813)

[6.3 Unblinding Procedures (Code break) 47](#_Toc106024814)

[7. CLINICAL INVESTIGATION POPULATION 48](#_Toc106024815)

[7.1 Eligibility criteria 48](#_Toc106024816)

[7.2 Recruitment and screening 48](#_Toc106024817)

[7.3 Assignment to investigation groups 49](#_Toc106024818)

[7.4 Criteria for withdrawal / discontinuation of subjects 49](#_Toc106024819)

[8. CLINICAL INVESTIGATION INTERVENTION 50](#_Toc106024820)

[8.1 Identity of the medical device under investigation 50](#_Toc106024821)

[8.1.1 Experimental Intervention (medical device) 50](#_Toc106024822)

[8.1.2 Control Intervention (standard/routine/comparator) 50](#_Toc106024823)

[8.1.3 Labelling and Supply (re-supply) 50](#_Toc106024824)

[8.1.4 Storage Conditions 50](#_Toc106024825)

[8.2 Discontinuation or modifications of the intervention 50](#_Toc106024826)

[8.3 Compliance with clinical investigation intervention 51](#_Toc106024827)

[8.4 Data Collection and Follow-up for withdrawn subjects 51](#_Toc106024828)

[8.5 Clinical investigation specific preventive measures 51](#_Toc106024829)

[8.6 Concomitant Interventions (treatments) 51](#_Toc106024830)

[8.7 Medical Device Accountability 51](#_Toc106024831)

[8.8 Return, Analysis or Destruction of the Medical Device 51](#_Toc106024832)

[9. CLINICAL INVESTIGATION ASSESSMENTS 52](#_Toc106024833)

[9.1 Clinical investigation flow chart/ table of clinical investigation procedures and assessments 52](#_Toc106024834)

[9.2 Assessments of outcomes 54](#_Toc106024835)

[9.2.1 Assessment of primary outcome 55](#_Toc106024836)

[9.2.2 Assessment of secondary outcomes 55](#_Toc106024837)

[9.2.3 Assessment of other outcomes of interest 55](#_Toc106024838)

[9.2.4 Assessment of safety outcomes 55](#_Toc106024839)

[9.2.5 Assessments in subjects who prematurely stop the clinical investigation 55](#_Toc106024840)

[9.2.6 Follow-up of the subjects after the regular termination of the clinical investigation 55](#_Toc106024841)

[9.3 Procedures at each visit 56](#_Toc106024842)

[9.3.1 Visit 0 – Screening and enrollment 56](#_Toc106024843)

[9.3.2 Visit 1 – Intervention 56](#_Toc106024844)

[10. SAFETY 57](#_Toc106024845)

[10.1 Definition and Assessment of (Serious) Adverse Events and other safety related events 57](#_Toc106024846)

[10.2 Adverse events categorization 58](#_Toc106024847)

[10.3 Documentation and reporting in Medical Device Category A clinical investigations 59](#_Toc106024848)

[10.3.1 Foreseeable adverse events and anticipated adverse device effects 59](#_Toc106024849)

[10.3.2 Reporting of Safety related events Reporting to the Sponsor 59](#_Toc106024850)

[10.3.3 Follow-up of (Serious) Adverse Events 60](#_Toc106024851)

[11. STATISTICAL METHODS 61](#_Toc106024852)

[11.1 Hypothesis 61](#_Toc106024853)

[11.2 Determination of Sample Size 61](#_Toc106024854)

[11.3 Readers 62](#_Toc106024855)

[11.4 Statistical criteria of termination of the investigation 63](#_Toc106024856)

[11.5 Planned Analyses 63](#_Toc106024857)

[11.5.1 Dataset to be analysed, analysis populations 63](#_Toc106024858)

[11.5.2 Primary Analysis 63](#_Toc106024859)

[11.5.3 Secondary Analysis 63](#_Toc106024860)

[11.5.5 Deviation(s) from the original statistical plan 63](#_Toc106024861)

[11.6 Handling of missing data and drop-outs 63](#_Toc106024862)

[12. QUALITY ASSURANCE AND CONTROL 65](#_Toc106024863)

[12.1 Data handling and record keeping / archiving 65](#_Toc106024864)

[12.1.1 Case Report Forms (CRF) 65](#_Toc106024865)

[12.1.2 Specification of source data and source documents 65](#_Toc106024866)

[12.1.3 Archiving of essential clinical investigation documents 65](#_Toc106024867)

[12.2 Data management 65](#_Toc106024868)

[12.2.1 Data Management System 65](#_Toc106024869)

[12.2.2 Data security, access and back-up 65](#_Toc106024870)

[12.2.3 Analysis and archiving 66](#_Toc106024871)

[12.2.4 Electronic and central data validation 66](#_Toc106024872)

[12.3 Monitoring 66](#_Toc106024873)

[12.4 Audits and Inspections 66](#_Toc106024874)

[12.5 Confidentiality, Data Protection 67](#_Toc106024875)

[12.6 Storage of related health data 67](#_Toc106024876)

[13. PUBLICATION AND DISSEMINATION POLICY 69](#_Toc106024877)

[14. FUNDING AND SUPPORT 69](#_Toc106024878)

[15. INSURANCE 69](#_Toc106024879)

[16. REFERENCES 70](#_Toc106024880)

[17. Appendices 74](#_Toc106024881)

# SYNOPSIS

| Sponsor/Sponsor-Investigator | Prof. Dr. Marios-Nikos Psychogios, M.D. (main contact person)  University Hospital Basel |
| --- | --- |
| Co-Principal-Investigators and US-Chair | Dr. Adam S. Arthur, M.D., M.P.H. (US Chair)  Semmes Murphey Clinic and University of Tennessee Health Sciences Center |
| Title | ProSPective evaluation of the dIagnostic accuracy of siNe spiN non-contrast flat-dEtectoR CT (FDCT) imaging for the detection of intracranial hemorrhage in Stroke patients – an open labelled, multicenter, non-inferiority comparison of FDCT to multi detector CT (MDCT) with blinded assessment of outcome events |
| Short Title | SPINNERS |
| Clinical Investigation Plan, Version and Date: | Version 2.0 29.04.2024 |
| Registration: | Clincialtrials.gov: NCT05458908 and SNCTP |
| Category and its rationale: | According to Swiss legislation (Human research act (HRA)^5^ and Art 6 Clinical Trial Ordinance-MD (ClinO-MD)^2^) the clinical investigation is categorized as a clinical investigation concerning a medical device class A2, since the medical device under investigation is CE-certified, commercially available in Switzerland and it will be used within its intended use. As the additional performance of the non-contrast syngo DynaCT Sine Spin may not be clinical routine in a subgroup of subjects and in these subjects associated with excess radiation (up to 2.5 mSV, according to internal measurements and recent publications)^3, 6^ ,the clinical investigation is rated as A2. No contrast-media will be applied for the study.  US medical device classification Class 2. Minimal risk to subject (up to 2.5 mSv according to internal measurements and recent publications) |
| Name of the MD,  Unique Device  Identification (UDI),  name of the  manufacture | ARTIS icono biplane angiography system, model no.: 11327600, software version VE20 or higher with syngo application software, model no.: 11327667, version VE20D or higher with syngo DynaCT Sine Spin head imaging protocol.  UDI-DI ARTIS icono: 04056869063317  UDI-DI syngo application software: 04056869124162  US 510(k) Number:  ARTIS icono K201156  syngo application software K190780 |
| Stage of development | Post-market stage |
| Background and rationale | Stroke is one of the leading causes of mortality and disability worldwide. Mechanical thrombectomy became the gold-standard for the treatment of acute ischemic stroke due to large-vessel occlusions (LVO) after the publication of five large landmark trials.^7, 8^ However, as was shown by a post-hoc meta-analysis of these five trials clinical outcome is highly associated with the time from hospital admission to reperfusion.^9^ One possibility to substantially shorten this time span is the implementation of a direct to angiography approach. In this workflow both imaging and subsequent EVT is done in the angiography suite using non-contrast flat detector CT (FDCT) for the exclusion of an intracranial hemorrhage.^10^ We and other workgroups were already able to show in large patient series that such workflows dramatically reduce intra-hospital time delays (median reductions of more than 30 minutes) and are associated with improved patient outcomes.^11-14^ This workflow is mainly suitable for suspected stroke patients with disabling symptoms (National Institute of Health Stroke Scale (NIHSS) ≥ 7), since in this patient population the likelihood of a treatable LVO is substantially higher compared to patients with lower NIHSS scores.^15, 16^  One of the biggest hurdles for a large-scale implementation of a direct to angiography approach up to now is the ability to differentiate between ischemic and hemorrhagic stroke with FDCT. In earlier generations of angiography equipment, image deterioration due to artefacts and reduced image quality near the skull and the skull base limited the use of FDCT as a triage tool.^17^ However, in a recent study we reported very high sensitivity and specificity for the detection of intracranial hemorrhage with FDCT.^18^ Recently Siemens Healthineers introduced the new ARTIS icono angiography system with a new non-contrast syngo DynaCT Sine Spin protocol FDCT (from here-on in the CIP FDCT and non-contrast syngo DynaCT Sine Spin FDCT will be used synonymously), which should improve the quality and soft tissue resolution of native cranial FDCT scans especially in the posterior fossa and skull base.  Therefore, we aim to evaluate if non-contrast syngo DynaCT Sine Spin FDCT is non-inferior to non-contrast multidetector CT (MDCT) for the detection of intracranial hemorrhages. |
| Objective(s): | The primary objective is to evaluate if non-contrast FDCT imaging is non-inferior compared to non-contrast MDCT for the detection and exclusion of intracranial hemorrhages. Non-contrast FDCT imaging will be deemed non-inferior if both the lower bounds of the 95% confidence interval (CI) of the sensitivity and of the specificity are above 95% (pre-defined non-inferiority margins), using MDCT as the reference standard.  The secondary objectives are to determine the sensitivity, specificity, positive predictive value, and negative predictive value of non-contrast FDCT imaging for the detection of intracranial hemorrhage. The sensitivity (including its 95%-CI) for the detection of an intracranial hemorrhage within the subset of patients presenting with an isolated infratentorial intracranial hemorrhage will be reported separately. A further secondary objective is to determine the interrater agreement for non-contrast FDCT imaging regarding the occurrence of intracranial hemorrhage. |
| Outcome(s): | The primary outcome is the occurrence of intracranial hemorrhages (yes vs no) as assessed by a blinded core-lab. The primary outcome will be used to calculate the sensitivity and specificity of non-contrast syngo DynaCT Sine Spin imaging for the detection of intracranial hemorrhages.  There are no secondary outcomes. |
| Design: | Cross-sectional non-inferiority investigation with prospective open label data collection and blinded endpoint assessment. |
| Inclusion/Exclusion criteria: | Inclusion criteria:   - Informed Consent as documented by signature or fulfilling the criteria for emergency or deferral of consent - Patients with symptoms suggestive of stroke ischemic (NIHSS ≥ 7) or hemorrhagic stroke with a cranial non-contrast MDCT and a feasible non-contrast syngo DynaCT Sine Spin FDCT within 4 hours - Patient presenting within 24 hours of last seen well - Patients presenting directly to the treating hospital (i.e. mothership patients) OR transfer patients with the indication for repeated imaging according to the standard operation procedures of the treating hospital - Age above 18 years - Agreement of treating physician to perform non-contrast syngo DynaCT Sine Spin FDCT   Exclusion criteria:   - Severe metal artefacts on initial MDCT imaging - Planned invasive interventions between MDCT and FDCT scan - Clinical deterioration between MDCT and FDCT scan (i.e. an increase of the NIHSS of more than 4 points) - Evidence of an ongoing pregnancy prior to enrollment. A negative pregnancy test before enrollment is required for all women with child-bearing potential |
| Measurements and procedures: | All subjects will undergo non-contrast cranial MDCT and non-contrast FDCT imaging with a maximal time span of four hours between the two scans. Only patients in whom no invasive procedure is planned or performed in between the scans can be enrolled. Subjects will be enrolled based on clinical features (symptoms of the stroke) and according to a pre-defined number of needed hemorrhagic and non-hemorrhagic stroke patients. They will be recruited in a ratio of 1:1. This will be secured over an online enrollment system in which the investigator has to enter if a patient has a hemorrhagic (and location of hemorrhage) or non-hemorrhagic stroke. All sites must maintain screening logs.  To ensure compliance with the Protocol and adequate acquisition technique of the FDCT and MDCT at the respective site the first five cases from each site will be deemed as lead-in cases. Cases can be excluded and deemed as Screen Failures if they show clear evidence of (a) operator failure (for example due to clear artifacts due to objects in the field of view, wrong positioning of head, wrong acquisition protocol, severe motion artifacts, missing reconstructions) or (b) technical failure due to missing calibration of the scanner. The ultimate decision to exclude cases is made by the Independent Core Lab (under the supervision of Prof David Liebeskind). In such a case it is the obligation of the Sponsor to perform retraining. Excluded cases will be replaced to ensure adequate power.  All non-contrast MDCT and FDCT scans will be rated by blinded readers, who were not involved in the original treatment or enrollment of the subjects on an individual basis in a core-lab (the core lab service will be provided by the Neurovascular Imaging Research Core, Prof. David Liebeskind, University of California Los Angeles, Los Angeles, USA). The readers will be experienced in neuroimaging. All scans will be reconstructed in a standardized matter (i.e., fixed slice thickness 5 mm, distance 3 mm and angles – orbitomeatal) and randomized prior to evaluation. All MDCT scans will be read by one reader with no time-limit, while all FDCT scans will be read by six readers with a time-limit of 90 seconds (only applicable to the decision hemorrhage positive or negative). The reader of the MDCT scan will not be part of the six readers who read the FDCT scans. Each of the six readers will read all FDCT scans completely independent from the other readers, and they will not share any information.  The ground truth (i.e., if an intracranial hemorrhage is present) will be defined based on the MDCT readings according to the following procedure. If the readings of the clinical reader and the core-lab are consistent, this will be deemed as ground truth. In case they are inconsistent (i.e., at the clinic it is rated as hemorrhage positive and in the core-lab as hemorrhage negative or vice versa) a second independent core-lab consisting of a highly experienced neuroradiologist (Prof. Jan Gralla, Inselspital Bern, Bern, Switzerland) will read the MDCT scan and review all available clinical data. Based on this information he will make a final ruling (i.e., define the ground truth for this patient, majority vote).  The FDCT readings of the six Core-Lab readers will be compared with the ground truth and based on this comparison they will be rated as true positive, true negative, false positive or false negative. These values will be calculated for each reader separately. In case most of the core-lab 1 readers (at least 5 out of 6) detects a hemorrhage on the FDCT scan, which according to the ground truth was hemorrhage negative the second core-lab will use all available clinical data to deduce if there is any evidence for the hemorrhage to have occurred between both scans. In this case the patient will be excluded from further analysis.  See table 1 for a detailed overview of all possible constellations.   \| Ground truth \| Core-Lab 1 \| Core-Lab 2 \| Final Result \| \| --- \| --- \| --- \| --- \| \|  \| FDCT \| Unblinded \| FDCT \| \| (+) \| (+) \| NA \| True (+) \| \| (-) \| (-) \| NA \| True (-) \| \| (+) \| (-) \| NA \| False (-) \| \| (-) \| (+) \| (-) \| False (+) \| \| (-) \| (+) \| (+) \| Hemorrhage occurred between scans \|   Table 1. Overview of possible outcomes  The results are used to calculate the sensitivity and specificity of the FDCT. |
| Interventions: | The examined intervention is a non-contrast cranial syngo DynaCT Sine Spin scan with an ARTIS Icono angiography system.  The intervention itself takes under 1 minute. The timespan from enrolment to the last investigation specific visit (the non-contrast syngo DynaCT Sine Spin) is 4 hours at max. |
| Control: | The control intervention is a non-contrast cranial MDCT (at least 64 slice) |
| Number of subjects with rationale: | The existing literature clearly documents MDCT as the gold standard for the detection of acute, spontaneous intracranial hemorrhage.^19^ Therefore the readings of the MDCT will be set as ground truth.  To calculate the sample size, hypothetical experiments were simulated. To account for variation, the diagnostic performance of hypothetical readers was varied around 99% for the sensitivity and around 98% for specificity. Rates were attributed randomly with equal chance. To represent “difficult cases”, i.e. patients who present with an isolated infratentorial hemorrhage we introduced a subpopulation of patients for whom the performance of raters was only 70% sensitivity. This subset was weighted in the final sample size calculations with 2 %.  Each sample size, n_i=1,..,19_ = 80,…,260 was evaluated by sampling 999 times n_i_ individual samples with a range of readers from 1 to 7.  If power is set at 90%, the number of readers at 6, and the drop-out rate at 5%, a total of **252 patients (**238 patients without drop-outs) must be recruited. The patients must be pre-selected in a ratio of hemorrhage positive to hemorrhage negative of 1:1, i.e., 126 patients are presenting with and 126 patients without intracranial hemorrhage. To ensure an enrollment in this ratio, a bucket design will be used. There will be three predefined buckets: (1) 126 acute ischemic (non-hemorrhagic) stroke patients, (2) 126 hemorrhagic stroke patients, which include (3) 14 patients with an isolated infratentorial or basal hemorrhage. |
| Duration of the investigation: | 36 months total  24 months enrollment period |
| Investigation schedule: | 25. October 2022: first patient-in (anticipated)  31. December 2024: last patient-out (anticipated) |
| Investigator(s): | Prof. Dr. Marios-Nikos Psychogios, M.D. (PI)  Dr. Adam S Arthur, M.D. (Co-PI and US Chair) |
| Investigational site(s) | Multicentric (up to 30 sites)  Up to 30 sites in Switzerland, United States of America, France, Finland and Spain |
| Statistical  considerations: | The primary analysis will be based on the full analysis set.  A generalized linear random-effects model with a logit link function will be fit. From the fixed-effect intercept of sensitivity and specificity, the 95%-Cis will be calculated applying the method of Wald. Difficult cases will be weighted with 2%. Sensitivity will be calculated based on the results of all core-lab readers of the non-contrast FDCT scans of all patients for which the ground truth was deemed to be hemorrhage positive. Specificity will be calculated based on the results of all core-lab readers of the non-contrast FDCT scans of all patients for which the ground truth was deemed to be hemorrhage negative. It will be tested whether both the lower bound of the 95%-CI of the sensitivity and of the specificity is higher than the margin of 95%.  All results will be reported in concordance with the STARD guidelines.^20^ |
| Compliance statement: | This investigation will be conducted in compliance with the CIP, the current version of the Declaration of Helsinki, ISO14155, ICH-GCP (as far as applicable) as well as all national legal and regulatory requirements. |

# ABBREVIATIONS

ADE Adverse Device Effect

AE Adverse Event

AHA/ASA American Heart Association/American Stroke Association

AIS Acute ischemic stroke

ASADE Anticipated Serious Adverse Device Effect

ASR Annual Safety Report

CA Competent Authority (e.g., Swissmedic)

CEC Competent Ethics Committee

CI Confidence Interval

CIP Clinical investigation plan

ClinO Ordinance on Clinical Trials in Human Research

ClinO-MD Ordinance on Clinical Trials with Medical Devices

CRF Case Report Form (pCRF paper CRF; eCRF electronic CRF)

DD Device Deficiency

DSMC Data Safety Monitoring Committee

FAS Full Analyse Set

FDCT Flat Detector Computer Tomography (will be used as a synonym for non-contrast syngo DynaCT Sine Spin)

GLMM Generalized linear mixed model

Ho Null hypothesis

H1 Alternative hypothesis

HRA Federal Act on Research involving Human Beings

IB Investigator’s Brochure

ICF Informed Consent Form

ICH Intracranial Hemorrhage

ICH-GCP International Council for Harmonisation – guidelines of Good Clinical Practice

ICL Imaging Core Lab

IFU Instruction for Use

ISF Investigator Site File

ISO International Organisation for Standardisation

ITT Intention to treat

IRB Institutional Review Board

LAR Legally authorized representative

LVO Large Vessel Occlusion

NIHSS National Institute of Health Stroke Scale

MedDO Medical Devices Ordinance

MD Medical Device

MDCT Multi detector Computer Tomography

MDR Medical Device Regulation (EU) 2017/745 of 5 April 2017

OEM Original Equipment Manufacturer

PI Principal Investigator

SADE Serious Adverse Device Effect

SAE Serious Adverse Event

SADE Serious Adverse Device Effect

SAH Subarachnoid hemorrhage

SAP Statistical Analysis Plan

SDV Source Data Verification

SNCTP Swiss National Clinical Trials Portal

SOP Standard Operating Procedure

USADE Unanticipated Serious Adverse Device Effect

# INVESTIGATION SCHEDULE

| **Schedule of assessments** | | | |
| --- | --- | --- | --- |
| **Visits** | | **0** | **1** |
|  |  | **Screening and enrollment** | **Intervention** |
| **Assessment** | **Time Window** | Admission | 0 – 4 hours after Non-contrast MDCT scan |
|  | **Method** | Clinical Visit | Intervention |
| Informed consent | | X^1^ |  |
| Patient demographics (i.e. age, gender, pre-existing conditions) | | X |  |
| National Institute of Health Stroke Scale | | X |  |
| Pregnancy test (only in women of childbearing potential) | | X |  |
| Patient logistics (i.e. time from onset to admission / imaging) | | X |  |
| Medical history (including medication) | | X |  |
| Non-contrast MDCT head scan^2^ | | X |  |
| Enrollment | | X |  |
| Non-contrast syngo DynaCT Sine Spin FDCT head scan | |  | X |
| AEs | | Only if procedure related up to 24 hours after procedure | |
| SAEs | | Only if procedure related up to 24 hours after procedure | |

1. Post-hoc consent if patient was not able to give consent at trial inclusion (according to national and applicable law)

2. For clarification the MDCT scan could have been done in an external hospital (in case of transfer patients) if the images of the MDCT are available at the PACS of the enrolling hospital and the quality was deemed to be sufficient by the enrolling physician and all other in- and exclusion criteria are met

AEs Adverse Events, FDCT Flat-Detector CT, MDCT Multi-Detector CT, SAEs Serious Adverse Events

# 1. INVESTIGATION ADMINISTRATIVE STRUCTURE

## 1.1 Sponsor, Sponsor-Investigator

Prof. Dr. Marios-Nikos Psychogios, MD (main contact person)

University Hospital Basel

Petersgraben 4

CH-4051 Basel

Roles: overall responsibility for initiation and conduct of the clinical investigation, clinical investigation design, data collection, management, analysis, interpretation of data and writing of the publication

## 1.2 CO-PI and US-Chair

Dr. Adam S. Arthur, MD, MPH

Semmes Murphey Clinic and University of Tennessee Health Sciences Center

6325 Humphreys Blvd

Memphis, TN 38129

USA

Roles: clinical investigation design, interpretation of data and writing of the publication

## 1.3 Principial Investigators (PIs) in Switzerland and abroad

Please find a comprehensive list of all participating investigators in Appendix A Principal Investigators.

Roles: responsible for the conduct of the investigation at their site

## 1.4 Statistician

Nikki Rommers, PhD

Department of Clinical Research

University Hospital Basel

Spitalstrasse

CH-4051 Basel

## 1.5 Laboratory

Not applicable

## 1.6 Monitoring Institution

Lead-Monitoring Institution:

Clinical Trial Unit Basel

Schanzenstrasse 55

CH-4031 Basel

US-Monitoring Institution:

Semmes Murphey Foundation

6325 Humphreys Blvd.

USA, Memphis, TN 38120

## 1.7 Data Safety Monitoring Committee (DSMC)

The DSMC is independent. None of the DSMC members will be involved in the enrollment of patients in this clinical investigation. This board shall provide recommendations to the Sponsor and Investigators regarding stopping/continuing enrolment in the clinical investigation. The DSMC role, reporting structure, meeting modalities etc. are defined and regulated in a separate DSMC charter. The DSMC will meet after enrollment of 25% (63 patients), 50% (126 patients), and 75% (189) of the patients. The DSMC consists of the following members:

Prof. Georgios Tsivgoulis , M.D., PhD, MSc

Chairman of the Second Department of Neurology

National & Kapodistrian University of Athens,

GRC-Athens 157 72

Prof. Dr. Antonia Zapf

Deputy Head of the Department of Medical Statistics

University Hospital Hamburg Eppendorf

Martinistraße 52, 20251 Hamburg

Prof. Robert Starke, MD

Department of Neurosurgery

Jackson Memorial Hospital

1611 NW 12^th^ Avenue, Miami, FL 33136

## 1.8 Primary Imaging Core Lab (ICL)

The objectives of the primary ICL are to provide an unbiased assessment of several imaging measures. All assessments will be made according to a Core-Lab manual, which is written and finalized prior to any reading. They are knowledgeable in the analysis of neuroradiological images and will be blinded, i.e., unaware of the patient`s treatment allocation and the clinical investigation results. The ICL will receive de-identified imaging data only. ICL results will overrule site/investigator assessments. The qualified institution as ICL is:

Neurovascular Imaging Research Core

Director Prof. David S Liebeskind, MD, FAAN, FAHA, FANA, FSVIN, FWSO

University of California LA

Los Angeles, California

United States of America (USA)

## 1.9 Secondary Imaging Core Lab (ICL)

The objective of the second ICL is to make a final ruling a) in case there is a disagreement in the clinical rating and the first core-lab rating of the MDCT or b) in case the majority of the first core-lab ratings (at least 5 out of 6) of the FDCT is hemorrhage positive and the ground truth is hemorrhage negative, to determine if the hemorrhage has occurred between both scans. The second ICL will be blinded to the rating of the core-lab 1 but not to the remaining clinical data. The second ICL defines the ground truth for all patients in whom the clinical rating and the rating of core-lab 1 of the MDCT are divergent (majority vote).

The second ICL will be performed by Prof. Jan Gralla, MD (Head of the Clinic for diagnostic and interventional Neuroradiology, University Hospital Bern), who is an internationally known Neuroradiologist with more than 20 years of experience in the assessment of cranial non-contrast images in stroke patients. All readings will be done in accordance with a core-lab manual, which will be written prior to any assessment. As the second ICL will not be blinded to any data (except to the readings of the first ICL), it is not a problem that Prof. Jan Gralla, MD might have been involved in the original enrollment of patients at the study site Inselspital Bern, Bern, Switzerland.

Prof. Jan Gralla, MD

Head of the Clinic for diagnostical and interventional Neuroradiology

University Hospital Bern

Freiburgsstrasse 18

CH-3010 Bern

## 1.10 Trial Management and Administrative Lead

Alex Brehm, PhD

Department of interventional and diagnostic Neuroradiology

Petersgraben 4, CH-4051 Basel

## 1.11 Data Management

Clinical Trial Unit Basel

Schanzenstrasse 55

CH-4031 Basel

# 2. ETHICAL AND REGULATORY ASPECTS

We acknowledge the ethical requirements in clinical research as they were outlined by Emanuel E et al.^21^ As most of the points are detailed in later parts of this clinical investigation plan (CIP), we will give in this section only a shortened statement with regard to the points (1) value of the research, (2) scientific validity, (3) fair subject selection, (4) favorable risk-benefit ratio, (5) independent review, (6) informed consent and (7) respect for the enrolled subject.

The value of the proposed clinical investigation lies in the validation of a new imaging modality for suspected acute stroke patients, which might allow in the future to triage severely affected stroke patients faster (studies point to a time gain of approximately 30 minutes).^11-13^ This might ultimately lead to better clinical outcomes in this often severely affected patient group, which would lower their burden of disease substantially.^9, 14, 22-25^ As stroke is still one of the leading causes of mortality and disability worldwide,^26-28^ this could have relevant effects on socioeconomic costs as well.^29^ (see section 3.1) The proposed clinical investigation will be the first investigation evaluating the non-contrast syngo DynaCT Sine Spin imaging protocol for the detection of acute, spontaneously occurring intracranial hemorrhage prospectively and with blinded outcome assessment, guaranteeing high validity of the findings. Furthermore, patients will be enrolled based mainly on clinical features (i.e., their symptoms), guaranteeing that the population enrolled within this clinical investigation, resembles the patient population which undergoes the procedure in everyday practice. The syngo DynaCT Sine Spin scan will be done as in standard clinical practice. This guarantees high external validity of our findings (see section 6). As pointed out subject selection will be done in close accordance with the patient population, which would undergo this procedure in everyday clinical practice (see section 7). Treatment delays due to the syngo DynaCT Sine Spin will be negligible, as the patient positioning on the angiography table is identical for the syngo DynaCT Sine Spin as for the following intervention. Therefore, the net treatment delay in patients not routinely undergoing syngo DynaCT Sine Spin is below 1 minute (time for acquisition of the sine spin FDCT is 7 seconds). In most of the subjects the second syngo DynaCT Sine Spin is clinical routine as it is often used to obtain a baseline status of the subject after transport to the angiography suite. This is done for example to rule out new or worsening hemorrhages prior to the intervention. In some subjects presenting with an intraparenchymal/intraventricular hemorrhagethe syngo DynaCT Sine Spin scan is not usually done in clinical routine. These subjects will receive minor extra radiation (up to 2.5 mSV) as in these patients the syngo DynaCT Sine Spin would not be done in clinical routine. ^6^

However, for evaluating bleeding detection it is of pivotal importance to include all kinds of bleedings to provide an evidence-based judgement on the diagnostic performance for the detection of intracranial hemorrhage. Although the subjects will have no personal gain from participating in this investigation, the gain for the society might be considerable due to improved triage of stroke patients (see section 3.7). The CIP will be independently reviewed by the ECs of all participating sites and an independent data safety monitoring committee (DSMC) will oversee the trial conduct. Informed consent must be obtained in all subjects in accordance with local legislation and standards (see section 2.7). Furthermore, prior to performing non-contrast syngo DynaCT Sine Spin the local investigator must obtain agreement from the treating physician to perform non-contrast syngo DynaCT Sine Spin in the subject at question. All subjects will be informed about the clinical investigation and have the right to withdraw from it at any point without giving any reason. This will not affect their treatment. In case of new information becoming available, which affects the subjects in any way, it will be promptly communicated to them through the local investigators.

Before the trial will be started, the protocol, the proposed patient information and consent form as well as other trial-specific documents will be submitted to a properly constituted Ethics Committee (EC) / Internal Review Board (IRB) in agreement with local legal requirements, for formal approval. Any amendment to the protocol will as well be approved (if legally required) by these institutions. The decision of the EC/IRB concerning the conduct of the trial will be made in writing to the Sponsor-Investigators before commencement of this trial. At each site the clinical trial can only begin once approval from all required authorities (for the site) has been received. Any additional requirements imposed by the authorities shall be implemented.

## 2.1 Registration of the investigation

The investigation is registered on clincialtrials.gov (NCT05458908). In addition, we will register the trial in national language on the SNCTP (via BASEC) platform.

## 2.2 Categorization of the investigation (Switzerland)

According to Swiss legislation (Human research act (HRA)^1^ and Art 6 Clinical Trial Ordinance-MD (ClinO-MD)^2, 4^) the clinical investigation is categorized as a clinical investigation concerning a medical device class A2, since the medical device under investigation is CE-certified in accordance with Art 13 MedDO, commercially available in Switzerland and it will be used within its intended use. Since the performance of the non-contrast syngo DynaCT Sine Spin is in a subgroup of the enrolled patients not clinical routine, and associated with minor excess radiation (up to 2.5 mSv according to internal measurements and recent publications)^6^, the clinical investigation is rated as A2.^4^ No contrast media will be applied due to the study.*See Appendix C- US Protocol Amendments

## 2.3 Competent Ethics Committee (CEC)

In Switzerland, the protocol will be submitted according to Swiss law and according to the procedures of Swissethics. EC approvals for foreign sites will be obtained by the responsible PIs according to country specific applicable law with the support of the CTU in Basel. The responsible PI at each site will ensure that approval from an appropriately constituted EC is sought for the clinical trial. No changes will be made to the protocol without prior Sponsor-Investigators and EC approval, except where necessary to eliminate apparent immediate hazards to patients.

The US chair is responsible for the adherence to all applicable national regulations and requirements including EC and institutional review board (IRB) approval in North America.

*See Appendix C- US Protocol Amendments

### 2.3.1 Reporting duties to the Competent Ethics Committee

Amendments are reported according to Art. 15 ClinO-MD (see also 2.10). No changes are made to the CIP without prior Sponsor and CEC approval, except where necessary to eliminate immediate hazards to subjects. Refer to chapter 10 for safety reporting.

The regular or premature end of the investigation as well as the interruption of the investigation is reported to the CEC within 15 days (within 24 hours if it is due to security reasons) (Art. 36 ClinO-MD). The reasons for a premature end or an interruption have to be explained.

A final report shall be submitted within one year after the regular end of the investigation and within 3 months after a premature end of the investigation (Art. 37 ClinO-MD).

The US chair is responsible for timely reporting to the CEC and IRB in the USA.

## 2.4 Competent Authorities (CA)

The MD used in this trial is commercially available and consequently conformity labelled. They will be used in accordance with the approved indications and instructions. Therefore, no approval from the Swiss competent authority Swissmedic is needed.

For sites in the USA and EU, approval will be obtained according to local requirements (if applicable). For the US this is the responsibility of the US Chair.

## 2.5 Ethical Conduct of the Trial

The investigation will be carried out according to the CIP and with principles enunciated in the current

version of the Declaration of Helsinki^30^, the European Regulation on medical devices 2017/745 (MDR)^31^, the Norms ISO14155, the ICH-guidelines of Good Clinical Practice (GCP)^32^ as applicable, the Swiss Human Research Act (HRA)^1^ and its Ordinances and Swiss regulatory authority’s requirements. The CEC and the CA will receive the Annual Safety Report (ASR) and interim reports and be notified about investigation stop/end in agreement with local requirements.

Sites abroad will be informed about trial stop/end in the same manner as ECs in Switzerland and will receive annual safety and interim reports for submission to their ECs. Abroad, the local PI is responsible for the adherence to all applicable national and local requirements.

The US chair will be informed about trial stop/end in the same manner as ECs in Switzerland and sites abroad and will receive annual safety and interim reports for submission to their ECs. In the USA the US chair is responsible for the adherence to all applicable national and local requirements.

## 2.6 Declaration of interest

The trial is funded by Siemens Healthineers AG. This is an academic investigator-initiated trial (IIT). Experts nominated by and employees of Siemens Healthineers AG were consulted during the protocol development, but the Sponsor-Investigator and the Co-PI had the final decisions to all suggestions. Siemens Healthineers AG will have no influence on data acquisition, data management, data analyses or scientific interpretation and publication of the results.

## 2.7 Patient Information and Informed Consent

Each institution must follow their institutional and national EC policy as well as local IRB policy (if applicable) for obtaining informed consent. All information sheets and consent forms will be submitted to the EC to be reviewed and approved. Legally correct consenting procedures must be guaranteed for every subject.

This clinical investigation will include mainly vulnerable patients in an emergency situation. All patients must have a severe stroke or symptoms suggestive of an intracranial bleeding. Most patients need an emergency treatment. As determined by the inclusion criteria, only patients with severe neurological deficits (National Institute of Health Stroke Scale (NIHSS) ≥ 7) or symptoms suggestive of an intracranial bleeding are eligible for this clinical investigation. Some of these patients may not be able to give informed consent. There is no alternative group of patients in whom this procedure could be studied, as we must ensure that it is studied in a comparable patient population to which it will be applied in the future. Otherwise, our results would have no external validity. Because we include mainly vulnerable patients in an emergency and due to the life-threatening situation, no delay to the enrolment procedure can be accepted. In patients capable of giving informed consent, informed consent must be obtained prior to enrollment of the patient. The enrolling physician will decide after consultation with the treating neurologist/neurosurgeon if the patient is able to give informed consent. If he/she is not able to give informed consent due to the severity of his neurological deficit and there is no declaration of intent available (i.e., advance directive) that he does not want to participate in clinical research, the patient will be included in the trial without prior informed consent by him-/herself or a next of kin/legally authorized representative (LAR) and recovery consent from the patients or his/her next of kin/LAR has to be obtained post-hoc. An independent physician will be consulted prior to enrolling the patient into the trial to ensure and confirm that the enrollment of the patient does not cause any harm or poses an excess risk to the patient. This will be documented on a specific form. The independent physician will also ensure, that there were no signs or symptoms present, suggesting the unwillingness of the patient to participate in this clinical investigation.

In all patients, who were enrolled without prior informed consent by him-/herself, the patient or their LAR/next of kin will be sought for consent at the earliest time post-procedure. If a subject is not capable of giving their informed consent, all reasonable efforts will be made to obtain informed consent from the subject’s legally authorized representative (for example, relative or caregiver). This includes cases where the subject dies during the procedure or shortly thereafter or when the severity of the subject’s condition prohibits them from providing informed consent.

The local investigator explains (either before enrollment if possible or at the earliest time post-procedure) to each subject or in case the subject is not capable of giving informed consent to their LAR/next of kin the nature of the investigation, its purpose, the procedures involved, the expected duration, the potential risks and benefits and any discomfort it may entail. Each subject or their LAR/next of kin is informed that the participation in the investigation is voluntary and that he/she may withdraw from the investigation at any time and that withdrawal of consent will not affect the subject`s subsequent medical assistance and treatment. The subjects or their LAR/next of kin are informed that he/she can ask any question, and consult with family members, friends, their treating physicians, or other experts before deciding about their participation in the investigation. Enough time is given to the subjects or if applicable their LAR/Next of kin. The subjects or their LAR/next of kin are informed that authorized individuals other than their treating physician may examine his/her medical records. All subjects or their LAR/next of kin are given a subject information sheet and a consent form describing the investigation and providing sufficient information for the subjects or their LAR/next of kin to make an informed decision about their participation in the investigation.

Please refer to Appendix Patient Information and Informed Consent for detailed information regarding the inclusion requirements and informed consent process.

*See Appendix C- US Protocol Amendments

## 2.8 Subject privacy and confidentiality

The Sponsor-PI affirms and upholds the principle of the subjects right to privacy and that they shall comply with applicable privacy laws. Especially, anonymity of the subjects will be guaranteed when presenting the data at scientific meetings or publishing them in scientific journals.

Individual subject medical information obtained as a result of this study is considered confidential and disclosure to third parties is prohibited. Subject confidentiality will be further ensured by utilising subject identification code numbers to correspond to treatment data in the computer files. All information (i.e., Name, Address, Date of birth etc.) that could identify the subject will be replaced by a code. People who do not have access to this key list cannot connect the information with the subject. The code list will never leave the University Hospital Basel. It will be protected with adequate encryption and will only be accessible to authorized persons. For data verification purposes, authorised representatives of the Sponsor-Investigators (e.g. monitoring institution) or an EC may require direct access to parts of the medical records relevant to the study, including subjects’ medical history.

*See Appendix C- US Protocol Amendments

## 2.9 Early termination of the Investigation

The Sponsor-Investigator may terminate the investigation prematurely according to certain circumstances, for example:

- ethical concerns,
- insufficient patient recruitment,
- when the safety of the patients is doubtful or at risk, respectively,
- alterations in accepted clinical practice that make the continuation of a clinical investigation unwise or
- early evidence of benefit or harm of the experimental intervention

**Stopping rules as defined by DSMC**

The DSMC will stop the investigation if the procedure shows harm according to the procedure laid down in the DSMC charter. Interim analysis regarding safety endpoints (SAEs and mortality) will be done after 63 (25%), 126 (50%), and 189 (75%) subjects were enrolled as defined in the DSMC charter.

**Stopping rules for the individual patient**

A trial patient or his/her next of kin/LAR can withdraw the consent to participate in the investigation at any time and without any declaration of reason. In this case the data collected until the time of withdrawal will be included for evaluation in order to not compromise the validity of the clinical trial or its results.^1^ This also applies if the patient dies before it has been possible to obtain consent or refusal or in the absence of a statement of wishes, but only if the consent of a next of kin/LAR has been obtained.^1^ These data will be kept coded. If a patient was included into the trial without prior consent from the patient and the patient or his/her next of kin/LAR refuses to give post-hoc consent all data from this patient must be erased. This patient will be replaced by another patient.

## 2.10 Clinical investigation plan amendments

Substantial amendments are only implemented after approval by the CEC (Art. 15 ClinO-MD). Amendments can only be submitted by the Sponsor-Investigator. For USA, substantial amendments will be provided after approval by the Swiss lead EC to the US chair who is responsible to obtain the approval by their local responsible EC.

All non-substantial amendments are communicated to the CEC together with the Annual Safety Report

(ASR) (Art. 15 ClinO-MD). The ASR shall include any deviations from the CIP that may have affected the rights, safety or well-being of the subject or the scientific integrity of the investigation (ISO14155).

*See Appendix C- US Protocol Amendments

## 2.11 Deviation from the Clinical Investigation Plan

The use of waivers from the CIP is prohibited (Annex XV, Chapter 2, Art. 3.10 MDR).

Under emergency circumstances, deviations from the CIP to protect the rights, safety and well-being of human subjects may proceed without prior approval of the Sponsor-Investigators and the EC. Such deviations shall be documented and reported to the Sponsor-Investigator and the EC as soon as possible. Otherwise, deviations from the CIP are not permitted.

All CIP deviations will be analysed by the Sponsor-Investigator and the US Chair of the Investigation and addressed with the local PI responsible for the CIP deviation. The Sponsor-Investigator will work together with the local PI on solutions to prevent CIP deviations in the future and also record in writing the results of these consultations. In case of repetitive CIP deviations an investigator can be disqualified from the investigation.

*See Appendix C- US Protocol Amendments

## 2.12 Pregnancy

Evidence of an ongoing pregnancy prior to randomization is an exclusion criterion. A negative pregnancy test must be obtained for all persons of childbearing potential prior to randomization. However, if a pregnancy emerges after the intervention, this will not result in the exclusion of the patient.

As the MDCT is **not** part of the clinical investigation it is at the discretion of the treating physician to perform the pregnancy test prior to the MDCT. However, if it is decided that the patient is a candidate for the trial a pregnancy test must be performed. Due to the possible time delay of up to four hours between the MDCT and the FDCT this is feasible.

# 3. BACKGROUND AND RATIONALE

## 3.1 Background and Rationale for the clinical investigation

Despite concerted efforts, stroke is still one of the leading causes of mortality and disability worldwide.^26-28^ Stroke can be divided into two main types: ischemic and hemorrhagic stroke. In severely affected patients, which will most likely be disabled or dead after the stroke - if not timely treated - roughly 80% of the strokes are caused by a vessel occlusion (i.e., ischemic strokes) and 20% are due to an intracranial bleeding (i.e., hemorrhagic stroke).

For acute ischemic stroke (AIS) patients due to a vessel occlusion, mechanical thrombectomy (MT) has substantially reduced the burden of disease. However, still roughly 50% of all AIS patients due to a large vessel occlusion (LVO are severely disabled after the stroke or die within 90 days after the stroke.^7, 8^ Since all neuroprotective trials so far have failed to show any beneficial effect in such patients^33, 34^, patient triage is an important area of inquiry due to the high time-dependency of the efficacy of MT.^9^ On average one minute delay in time to treatment causes the irreversible loss of 1.9 million neurons, 14 billion synapses and 12 km of myelinated fibers.^35^ Especially the door to puncture time is an independent variable for patient outcome as was shown in an analysis of 6,756 AIS patients with an LVO. Among every 1,000 patients treated, every 15-minute decrease in door-to-puncture time was associated with 21 (95%-CI 8 – 34) more patients discharged to home, 18 (95%-CI 4 – 31) more patients having freedom from disability and 22 (95%-CI 7 – 37) more patients having functional independence at discharge. It was further associated with 15 (95%-CI 4 – 26) fewer in-hospital deaths or patients being discharged to a hospice.^24^ These results were further validated by other study groups.^9, 22, 23^ Reducing time-delays in AIS patients has also important implications on a socioeconomic level as was recently shown by Kunz et al. They concluded based on the data of 7 large randomized controlled trials (RCTs) that every 10 minutes of earlier treatment result in an average gain of 39 days (95% prediction interval 23 – 53) of disability free life, translating to a reduction of $10,915 (95% prediction interval $5,928 - $15,365) of life-time healthcare costs.^29^

As there are still no reliable pre-hospital tests for the detection of an LVO or for the differentiation of ischemic or hemorrhagic stroke, the optimization of intra-hospital pathways is as of today one of the most promising research topics in stroke treatment.^16, 36^ As of now, the optimized conventional workflow consists of a rapid clinical assessment of the patient, followed by diagnostic imaging with multi-detector CT and CT angiography (MDCT and MDCTA) in the CT room and MT of eligible patients in the angiography suite. This workflow leads in highly trained centers to a door-to-groin time of approx. 60-70 min.^37^ Logistical circumstances such as patient positioning and transport to separate rooms hinder further time reductions.

A potential solution to this problem is to do both imaging and subsequent MT in the angiography suite using non-contrast syngo DynaCT Sine Spin (FDCT) for the exclusion of intracranial hemorrhage and flat detector CT angiography (FDCTA) or digital subtraction angiography for diagnosis of LVO. This would omit the need for another stop in the MDCT room and allow for direct transfer of the patient to the treatment room.^10^ We and other workgroups were already able to show in large patient cohorts that this optimized “direct to angiography” workflow can reduce door to groin times to under 30 minutes.^11-13^ These results were confirmed by our recent meta-analysis.^38^

However, the widespread implementation of this direct to angiography workflow has been impended by the difficulty of differentiating between ischemic and hemorrhagic stroke with FDCT.^39, 40^ As it is not possible to differentiate between ischemic and hemorrhagic stroke without brain imaging, it is of pivotal importance that hemorrhage exclusion can be performed safely and reliably with native FDCT imaging as well. A missed intracranial hemorrhage, i.e. an hemorrhagic stroke (which make up between 15 and 20% of all severely affected stroke patients according to an analysis of the Swiss Stroke Registry) could have potentially dramatical (and lethal) consequences due to different treatment strategies.^41^ Up till now this was only evaluated in retrospective studies, which showed very high sensitivity and specificity in the detection of intracranial hemorrhage with the last generation of FDCT.^6, 18^ However, prospective high-quality studies with large patient cohorts on this topic are still missing. We further did not find any ongoing studies on clinicaltrials.gov or within the WHO trial registry.

Therefore, we aim with this study to investigate if non-contrast syngo DynaCT Sine Spin imaging is non-inferior to non-contrast MDCT imaging regarding its sensitivity and specificity for the detection of intracranial hemorrhages. Non-contrast syngo DynaCT Sine Spin imaging will be deemed non-inferior if the lower bound of the 95%-confidence interval (95%-CI) of the sensitivity is above 95% and the lower bound of the 95%-CI of the specificity is above 95% (pre-defined non-inferiority margin). If non-inferiority could be established, this would guarantee for a safe triage of stroke patients. We are confident, that if our study shows non-inferiority of non-contrast syngo DynaCT Sine Spin imaging it will pave the way for the widespread implementation of the above described direct to angiography workflow, allowing more AIS patients to live a self-sustained, independent life after their stroke. Due to the high burden of disease any measurement of improvement is of high need. Conversely if our study fails to establish non-inferiority of non-contrast syngo DynaCT Sine Spin imaging for the detection of intracranial hemorrhage, it might prevent patients from undergoing harmful treatments.

## 3.2 Identification and description of the Investigational Medical Device

The following CE-marked medical device will be used in the clinical investigation:

ARTIS icono biplane angiography system and syngo application software with syngo DynaCT Sine Spin 3-D head imaging protocol.

| Product/Trade Name: | Software version: | Model: | UDI-DI: |
| --- | --- | --- | --- |
| ARTIS icono biplane | VE21 or higher | 11327600 | 04056869063317 |
| syngo application software | VE21D or higher | 11327667 | 04056869124162 |

| Manufacturer: | Facility: |
| --- | --- |
| Siemens Healthcare GmbH  Henkestr. 127  91052 Erlangen, Germany | Siemens Healthcare GmbH Advanced Therapies  Siemensstr. 1  91301 Forchheim, Germany |

*See Appendix C- US Protocol Amendments

Single Registration Number (SRN): DE-MF-000006122.

There are no deviations from the original CE-marked instructions for use.

**ARTIS icono:**

Intended use: ARTIS, as a family of dedicated angiography systems generating fluoroscopic and radiographic X-ray images, is intended for diagnostic imaging, interventional and minimally invasive therapy.

Its use is indicated for image guidance in non-vascular, vascular, cardiovascular, neurovascular, minimal-surgical, and surgical procedures.

The ARTIS system includes also software options which allow the reconstruction of two-dimensional images acquired with rotational angiography into a three-dimensional image format, as well as options supporting diagnosis, planning, guidance and treatment follow-up.

Indications: In general, all clinical indications of angiography- and fluoroscopic-based procedures within the intended use are applicable for this device.

Contra-indications: For this product there are currently no known contra-indications. However, contra indications for angiography- and fluoroscopy- based procedures apply.

The final decision for use of the medical device in a certain application is made by the physician based on his/her medical knowledge and the risks of the cases involved.

Patient target group(s): The system can be used on all patients, from newborn to geriatric. Patient

weight is limited to the specification of the patient table.

Intended users:

Operator profile: The usage of the system requires specific technical and medical knowledge and skills regarding, at a minimum, radiation protection, safety procedures and patient safety. Those using or working with the system must have acquired such knowledge and skills during their curriculum.

Equipment training: Application training is delivered according to the handover contract. It is mandatory to follow such application training delivered by Siemens Healthineers before any use of the system. The follow-up training, which is necessary due to change of personnel, is in the responsibility of the operator of the system. Any additional training can be requested from Siemens Healthineers.

Device materials in contact with body tissues and/or fluids: Device is not in touch with the human body. The patient mattress is covered with sterile disposable sheets for infection control before patients are placed on the device. The patient may get in touch with the system while being moved onto the table which represents a minor contact before or after the interventional procedure. The materials of the devices and its accessories with a direct contact with skin (skin contact, < 3 hours) are biocompatible according to ISO 10993. The user controls tableside are also covered with sterile drapes for infection control.

**syngo application software:**

Intended use: The syngo Application Software is a medical software for real-time viewing,

image manipulation, 3D-visualization, communication, and storage of medical images and data on exchange media. It can also be configured within a network to send and receive DICOM data. It is used for diagnostic image viewing and post processing and for viewing and post processing during interventional procedures.

It is not intended to be used with digital mammography images.

syngo Application Software provides image guided solutions in the operating room for image guided surgery (by Image Fusion and by navigation systems), image guided solutions in interventional cardiology and electrophysiology and image guided solutions for interventional oncology, radiology and neuroradiology.

syngo Application Software can also be configured with a variety of syngo or Windows-based software options, which are intended to assist the physician in diagnosis, treatment planning and treatment control. It includes commercially available post-processing techniques and OEM options.

The syngo Application Software is a software only medical device. It defines minimum requirements to the hardware it runs on, which can be a standalone workstation or part of another medical device.

Indications: In general, all clinical indications of angiography- and fluoroscopic-based procedures within the intended use are applicable for this device.

Contra-indications: For this product there are currently no known contra-indications.

Intended purpose: The final decision for use of the medical device in a certain application is made by the physician based on his/her medical knowledge and the risks of the case involved.

Patient target group(s): The system can be used with patients which are newborn to geriatric.

Intended users:

Operator profile: The usage of the software requires specific technical and medical knowledge and skills regarding, at a minimum, safety procedures and patient safety. People using the software must have acquired such knowledge and skills during their curriculum.

Application training: Application training is delivered with the software installation according to the handover contract. It is mandatory to follow such application training delivered by Siemens Healthineers Representative before any use of the system. The follow-up training, which is necessary due to change of personnel, is in the responsibility of the operator of the system. Any additional training can be requested from Siemens Healthineers.

## 3.3 Preclinical evidence

N/A since all medical devices including software are CE marked and are used according to the approved intended use.

*See Appendix C- US Protocol Amendments

## 3.4 Clinical Evidence to Date

### 3.4.1 Systematic review: Search strategy

We conducted a systematic review of the existing evidence on bleeding detection with cranial non-contrast syngo DynaCT Sine Spin imaging and cranial non-contrast FDCT imaging in general. We searched Embase (via Elsevier), Medline (via Ovid), and the Cochrane Central Registry of Controlled Trials (CENTRAL) (date of last updated search March 23, 2022). The latter includes the trial registries clinicaltrials.gov and the World Health Organization International Clinical Trials Registry Platform (WHO ICTRP) search portal. Search strings around the concept flat detector CT, cone beam CT, head and cranial were composed of database-specific subject headings (where applicable) and text word synonyms. The search was confined to the time period from 2016 till now (January 2022) since the triage of stroke patients with FDCT was first described in 2017.^10^

### 3.4.2 Clinical evidence to Date

We screened 4,950 records, out of which one evaluated non-contrast syngo DynaCT Sine Spin for the detection of intracranial haemorrhage.^6^ Furthermore, we found a few publications evaluating non-contrast cranial FDCT imaging in general for bleeding detection.^18, 42, 43^ The two most recent publications found very high sensitivities for the detection of intraparenchymal or intraventricular haemorrhage (97-100%) but only moderate to high sensitivity for the detection of subarachnoid haemorrhages, especially in the posterior fossa.^6, 42^

However, all these studies had important methodical issues. Most importantly, only a minority of patients came from the most relevant patient group: suspected stroke patients presenting with a spontaneous intraparenchymal and/or intraventricular haemorrhage. They mainly used surrogate patients, as they included patients which developed an intraparenchymal and/or intraventricular haemorrhage after an intervention. Findings from these patients cannot be generalized to patients presenting with a spontaneous intraparenchymal and/or intraventricular haemorrhage as haemorrhage location, size and distribution might differ substantially. An analysis of the Swiss Stroke Registry of patients from the University Hospital Basel showed that in our targeted patient population (suspected acute stroke patients with an NIHSS ≥7) 16% presented with such a spontaneous haemorrhage. Secondly, the studies did not define the patient population prospectively but rather used all patients available for their analysis. Therefore, the reported sensitivities and specificities could not be generalized to our targeted patient population (patients presenting with symptoms suggestive of a severe acute ischemic stroke or an intracranial bleeding). Third, in some of their cases an FDCT which was done prior to an intervention was compared to an MDCT which was done after the intervention. In these cases, it is not always feasible to deduce, if a bleeding occurred due to the intervention or was missed in the first scan. Also, due to the retrospective nature of the studies selection bias could not be ruled out.

## 3.5 Justification for the design of the clinical investigation

Clinical evidence based on the so far conducted studies is insufficient due to multiple reasons:

a) the most important patient group (suspected stroke patients with a spontaneous intraparenchymal and/or intraventricular hemorrhage) was underrepresented in the aforementioned studies,

b) the studies were retrospective with unclear patient enrollment criteria,

c) the studies had low sample sizes and

d) they used different (outdated) systems for the acquisition of cranial FDCT images.

To address all these issues, we propose performing a cross-sectional non-inferiority investigation with prospective open label data collection and blinded endpoint assessment. Using this design enables us to generate high quality data for the estimation of the sensitivity and specificity of non-contrast syngo DynaCT Sine Spin imaging for the detection of intracranial hemorrhages. It will allow clinicians in the future to make evidence-based decisions regarding the ability of cranial FDCT imaging to detect intracranial hemorrhages in suspected acute ischemic stroke patients. To ensure high external validity of our findings, the enrolled patient population will closely assemble the patient population undergoing cranial FDCT imaging in the future.^11, 12^

Non-contrast syngo DynaCT Sine Spin imaging was chosen as it is already used in clinical practice for the triage of suspected stroke patients. It will be used in the same way as in clinical routine. We will not deviate from its intended use and there will be no deviation from the imaging protocols already used in clinical routine for the detection of intracranial hemorrhages. The main endpoints will be the sensitivity and specificity of non-contrast syngo DynaCT Sine Spin imaging for the detection of intracranial hemorrhage.

## 3.6 Explanation for choice of comparator/ground truth

To calculate the sensitivity and specificity of non-contrast syngo DynaCT Sine Spin imaging a ground truth must be defined. The ground truth will be defined based on the MDCT images from the same patient. MDCT was chosen as the reference standard for the following reasons:

(1) It is standard of care in most hospitals worldwide for the detection of intracranial hemorrhages in acute stroke patients.^19, 44^

(2) It was identified as the gold standard for the detection of intracranial hemorrhages by the American Heart Association/American Stroke Association (AHA/ASA).^19, 44^

(3) It has very high sensitivities for the detection of intracranial hemorrhages. Recent studies yielded sensitives of 99.7% to 100% for the detection of subarachnoid hemorrhages (SAHs), even in non-academic centers.^45-47^

## 3.7 Risk evaluation (Risk-to-Benefits rationale)

Anticipated adverse device effects and residual risks associated with the MD and the procedures involved in its use:

**Ionizing radiation:**

Anticipated adverse device effects include risks related to effects of ionizing radiation. Biological hazards of ionizing radiation include deterministic effects like skin burns (erythema), cataracts, (permanent) epilation, (delayed) skin necrosis and death. Stochastic hazards of ionizing radiation like x-rays include genetic defects that can lead to cancer or hereditary diseases. The radiation dose typically applied during the intervention in this clinical investigation is related to the performance of a syngo DynaCT Sine Spin non-contrast scan protocol for the detection of intracranial bleeding. The typical effective dose during this scan is up to 2.5 mSv (according to internal measurements and recent publications).^3, 6^

The risks related to ionizing radiation can be minimized by reducing the X-ray exposure to the necessary minimum. This is done by following the ALARA principle and by careful and systematic application of protection measures for the patient, the operator and the clinical staff which are described in detail in the instructions for use, section 3.16.

**Skin irritations:**

Skin irritation may occur due to long-term skin contact especially in the presence of chemical residues e. g. that of cleaning / disinfection substances. This risk is inherent to any medical procedure and patient contact is limited to sterile disposable sheets and cleaning instructions are provided in the instructions for use.

**Pressure necrosis:**

Pressure necrosis may also occur while the patient is in the examination position. The patient table and patient positioning devices allow patient positioning that reduces the risk of pressure necrosis. During long examinations, trained personnel is required to minimize the risk of pressure necrosis by adequate means e. g. decubitus prophylaxis while the patient remains in examination position. As the intervention itself the non-contrast cranial syngo DynaCT Sine Spin does only take 7 seconds for acquisition of the images and a maximum timespan of 3 minutes for the conduct including patient positioning, pressure necrosis due to the FDCT scan is highly unlikely. Furthermore, in case of an ischemic stroke due to a vessel occlusion or another treatable cause of stroke, the treatment itself will take far longer than the scan itself.

**General risks associated with the medical device:**

Further risks associated with the device include typical risks associated with medical imaging equipment, e. g. collisions during system movements, or system failures and are described in detail in the instructions for use. Precautions and warnings as well as preventive measures are implemented and also described in the instructions for use.

Although the subjects in our investigation have no direct clinical benefit from participating in this clinical investigation, it might be highly relevant for future suspected acute ischemic stroke patients. As outlined above the demonstration of adequate diagnostic power of non-contrast syngo DynaCT Sine Spin for the detection of intracranial hemorrhage will very likely lead to a more widespread implementation of direct to angiography approaches. In 2016 MT was performed in 27,576 patients in Europe and projections estimate that roughly 200,000 patients were eligible for MT (numbers taken from the 2016 European Stroke Organization, European Academy of Neurology and Stroke Alliance for Europe survey), highlighting the outstanding high patient numbers, who could potentially profit from this clinical investigation.

The risk to subjects in our investigation is minimal. As in our investigation all patients undergo both MDCT and FDCT imaging for the detection of intracranial hemorrhage, there is no foreseeable scenario in which an important diagnosis (i.e., if there is an intracranial hemorrhage) would be overseen due to enrollment into the investigation. All clinical decisions should be based on the initial MDCT scan and not on the FDCT scan. Furthermore, only patients in whom no invasive procedure for immediate treatment is planned between both scans are eligible, ruling out the risk of treatment delay due to participation in the study. The performance of a syngo DynaCT Sine Spin scan is clinical routine in most of the enrolled subjects. In subjects presenting with an acute ischemic stroke (50% of the subjects) or a SAH (5-10% of the subjects) a baseline syngo DynaCT Sine Spin scan prior to the intervention is done in clinical routine on a case-to-case basis to rule out the occurrence of new bleedings or worsening of the SAH. In subjects presenting with an intraparenchymal/intraventricular hemorrhage (approximately 40-45% of the subjects) the FDCT scan is not always done in clinical routine.

In subjects who would normally not undergo a syngo DynaCT Sine Spin scan, there is a foreseeable risk due to minor excess radiation (up to 2.5 mSv according to measurements from Siemens Healthineers and recent publications).^3, 6^ This is like the average yearly radiation from artificial sources. Deterministic radiation damage is very unlikely since the average dose of the used protocol is far below established thresholds. This statement extends to the skin entrance dose. Internal safety systems of the MD under investigation, prevents any deterministic radiation damage.

Treatment delays due to the syngo DynaCT Sine Spin scan will be negligible, as the patient positioning on the angiography table is identical for the syngo DynaCT Sine Spin as for the following intervention. Therefore, the net treatment delay in patients not routinely undergoing syngo DynaCT Sine Spin is under 1 minute (time for acquisition of the syngo DynaCT Sine Spin is 7 seconds).

## 3.8 Justification of the choice of the investigation population

The diagnostic accuracy of the studied procedure could only be evaluated in a comparable patient population, which will be subjected to this imaging modality in the future. An analysis of data from the Swiss Stroke Registry has shown that in other patient populations the frequencies, sizes and distributions of intracranial hemorrhages are considerably different.

Therefore, the investigation population will consist only of patients with symptoms suggestive of a severe life threatening acute ischemic stroke or symptoms suggestive of an intracranial hemorrhage. They will be identified based on clinical symptoms as it is done in clinical practice. Only patients with severe neurological deficits (NIHSS≥7) or symptoms suggestive of an intracranial hemorrhage are eligible. These criteria were chosen as in clinical practice these patients are most likely to be triaged with a direct to angiography approach.^11, 12^

Consequently, this investigation will include mainly vulnerable patients. As specified by the inclusion criteria, only patients with severe neurological deficits (NIHSS ≥7) or symptoms suggestive of an intracranial hemorrhage (for example reduced level of consciousness) are eligible. Some of these patients may not be able to give informed consent. Furthermore, the underlying disease may require emergency treatment including immediate therapeutic decisions under life threatening time pressure. There is no alternative group of patients capable of judgement, with the same life-threatening condition and with such a severe deficit in whom this diagnostic procedure could be studied and where inclusion in such a trial could be justified. Because we include mainly vulnerable patients in an emergency situation and due to the life-threatening situation, no delay to the enrolment procedure can be risked. We will obtain informed consent from the patient if he/she is capable of giving informed consent. The enrolling physician will decide after consultation with the treating neurologist if the patient is able to give informed consent. If he/she is not able to give informed consent due to the severity of his/her neurological deficit, the patient will be included in the trial without prior informed consent by him-/herself or a next of kin/LAR and recovery consent from the patients or his/her next of kin/LAR has to be obtained post-hoc. An independent physician will be consulted prior to enrolling the patient into the trial to ensure and confirm that the enrollment of the patient is in his/her best interest. Each institution must follow their institutional and national EC policy as well as local IRB policy (if applicable) for obtaining informed consent. For details about patient information and informed consent see section 2.7.

# 4. CLINICAL INVESTIGATION OBJECTIVES

## 4.1 Overall Objective

The overall objective is to evaluate the diagnostic ability of the non-contrast syngo DynaCT Sine Spin for the detection of intracranial hemorrhages.

## 4.2 Primary Objective

The primary Objective is to evaluate if non-contrast syngo DynaCT Sine Spin imaging is non-inferior for the detection of intracranial hemorrhages compared to non-contrast MDCT imaging. It will be deemed non-inferior if the lower bound of the 95%-CI of the sensitivity is above 95% and the lower bound of the 95%-CI of the specificity is above 95% for the detection of intracranial hemorrhages (pre-defined non-inferiority margin).

## 4.3 Secondary Objectives

The secondary Objectives are to determine:

- the positive predictive value of FDCT for the detection of intracranial hemorrhages.
- the negative predictive value of FDCT for the detection of intracranial hemorrhages.
- the interrater agreement of FDCT with regard to the occurrence of intracranial hemorrhage

## 4.4 Safety Objectives

The safety of non-contrast syngo DynaCT Sine Spin imaging will be evaluated by documenting all device related AEs and SAEs, which occur during the procedure and within a safety-period of up to 24 hours after the procedure.

# 5. CLINICAL INVESTIGATION OUTCOMES

## 5.1 Primary Outcome

The primary outcome is the occurrence of intracranial hemorrhages (yes vs no) as assessed by a blinded core-lab. The primary outcome will be used to calculate the sensitivity and specificity of non-contrast syngo DynaCT Sine Spin imaging for the detection of intracranial hemorrhages.

## 5.2 Safety Outcomes

All procedure and device related AEs and all Device Deficiencies will be evaluated until 24 hours after the procedure.

# 6. CLINICAL INVESTIGATION DESIGN

## 6.1 General clinical investigation design and justification of design

The clinical investigation is designed as a cross-sectional, non-inferiority trial with prospective open label data collection and blinded endpoint assessment. The intervention to be studied is non-contrast syngo DynaCT Sine Spin. The intervention will be done as in standard of care setting.

The patient population consists of patients with a suspected severe acute ischemic stroke (NIHSS at admission ≥ 7 and time from last seen well maximum 24 hours) or with symptoms suggestive of an intracranial haemorrhage (for example reduced consciousness). All patients will undergo first non-contrast MDCT imaging and then afterwards non-contrast syngo DynaCT Sine Spin imaging. As the study is designed to be cross-sectional, there is no control group. All patients will undergo the same procedures. Only patients in whom no invasive intervention is planned between both scans and in whom it is feasible to perform both scans within a time-period of up to four hours can be enrolled. This time span however is assumed to be far shorter in many patients (especially in mothership patients; most likely under 30 minutes). The total duration of the intervention itself will be 7 seconds scan time and up to 1 minute procedure time (not including positioning of the patient). Patient positioning is done independently from the participation in the proposed clinical investigation. The total duration of the clinical investigation for each patient will be up to four hours from enrolment to the last assessment. There is no further follow-up visit planed after the sine-spin non-contrast FDCT.

Patients will be enrolled after the non-contrast MDCT and best medical therapy. This approach was chosen as enrolment prior to the non-contrast MDCT, and best medical therapy would lead to inacceptable time delays in this patient cohort. Based on clinical symptoms and imaging findings of the non-contrast MDCT patients can be enrolled in one of three buckets: (1) acute ischemic stroke patients, (2) patients with an intracranial haemorrhage which has supratentorial portions and (3) isolated infratentorial or basal intracranial haemorrhages (including perimesencephalic SAH). In total 252 patients will be enrolled (for justification of sample size see section 11) and they are distributed over the buckets in the following proportions:

- Bucket 1 (acute ischemic stroke patients) 126 patients
- Bucket 2 and 3 (haemorrhage patients) 126 patients including 14 patients in bucket 3 (isolated infratentorial or basal intracranial haemorrhage including perimesencephalic SAH)

Local investigators must keep a screening log.

The primary objective (sensitivity and specificity of non-contrast syngo DynaCT Sine Spin imaging for the detection of intracranial haemorrhage) will be assessed by blinded readers in an imaging core-lab. All readers will be blinded to all clinical characteristics and will not be involved in the treatment or enrolment of patients. Prior to the core-lab assessment all scans will be anonymized, reconstructed in a standardised way (fixed angles and slide sizes) and randomized. All readings will be noted on standardized forms.

The ground truth (i.e., if an intracranial haemorrhage is present or not) will be defined based only on the MDCT scans according to the following procedure: If the reading of the clinical reader (the enrolling physician at the participating site) and the reader from the first blinded core-lab (provided by the Neurovascular Imaging Research Core, Prof. David Liebeskind, University of California LA, Los Angeles, California, USA) is consistent this will be deemed as ground truth. If they are inconsistent a second core-lab (provided by Prof. Jan Gralla, Inselspital Bern, Bern, Switzerland) will make a final ruling (majority vote). The second core-lab can use all available clinical information but will be blinded to the results of the reading from core lab one. The following table illustrates the definition of the ground truth:

| Modality | Reader | Analysis |
| --- | --- | --- |
| MDCT | Clinical Reader | Agreement: ground truth |
|  | Core-Lab 1 | Disagreement: Unblinded expert reader in 2^nd^ corelab determines ground truth (majority vote) |

Table 1: Definition of the ground truth

The FDCT readings of the six core-lab readers will be compared with the ground truth and based on this comparison they will be rated as true positive, true negative, false positive or false negative. This will be done separately for each reader. In case most of the first core-lab readers (at least 5 out of 6) detect a hemorrhage on the FDCT scan, which according to the ground truth was no present on the MDCT scan, the second core-lab will use all available images and clinical data (unblinded) to decide if there is any evidence for the hemorrhage to have occurred between the MDCT and FDCT scans (always a possibility in patients with a stroke, e.g., without a hemorrhage on MDCT, treated with iv-lysis and then with a hemorrhage on FDCT as a complication of intravenous lytic treatment). In this case the patient will be excluded from further analysis. See table 2 for a detailed overview of all possible constellations:

| Ground truth | Core-Lab 1 | Core-Lab 2 | Final Result |
| --- | --- | --- | --- |
| MDCT | FDCT | FDCT | FDCT |
| (+) | (+) | NA | True (+) |
| (-) | (-) | NA | True (-) |
| (+) | (-) | NA | False (-) |
| (-) | (+) | (-) | False (+) |
| (-) | (+) | (+) | Hemorrhage occurred between scans |

Table 2. Overview of possible outcomes for the FDCT readings

Based on the results the sensitivity and specificity of non-contrast syngo DynaCT Sine Spin for the detection of hemorrhages will be calculated.

We chose this design approach for the following reasons:

1. The only way to benchmark the diagnostic performance of non-contrast syngo DynaCT Sine Spin imaging for the detection of intracranial haemorrhage is to compare it to the gold standard, which is cranial non-contrast MDCT imaging.^19, 44^ Therefore, it is of pivotal importance that scans of the same person, which were acquired under comparable conditions are used. This is ensured by the facts that (a) only patients in whom no invasive procedure between both scans is planned can be enrolled and (b) that the time period between both scans is under four hours. Using these criteria, it is highly unlikely, that new bleeding events occur between both scans. However, to account for the very low possibility of an early bleeding due to intravenous lysis, which would occur after the MDCT but before the FDCT, a second core-lab was implemented to identify these cases.

2. Although patients will be enrolled based mainly on clinical features, we defined a required number of patients (n = 14) with bleedings which are according to current literature harder to detect on predecessor FDCT generations than on MDCT, i.e. isolated bleedings in the posterior fossa / isolated infratentorial hemorrhages.^39, 48^ This approach was chosen to estimate the diagnostic performance of non-contrast syngo DynaCT Sine Spin imaging also in challenging, rare cases. It therefore allows a broader estimate of its ability for the detection and exclusion of intracranial hemorrhages. However, for the final analysis these cases will have a lower weight as they would have if they were count in by their proportion (14 isolated infratentorial bleedings out of 126 bleeding cases: 11%). In the final analysis they will contribute to the estimate of the sensitivity for the detection of intracranial hemorrhage of FDCT with a fixed weight of 2% (please refer to section 11.5 for a detailed description of the primary analysis). This weight which is lower than their share within the sample size was chosen as from the principal investigators and the US chairs clinical experience (including over 30 years of attending work in neuroradiology/neurosurgery) these cases are very rare (< 1%) in the investigated population and rarely present with symptoms, which would lead to a direct to angiography approach. To further validate their estimated prevalence in the patient group of question (patients with symptoms suggestive of an acute ischemic stroke), we did an analysis of all suspected stroke cases presenting to the University Hospital Basel in the years 2015 to 2019. The data was derived from the Swiss Stroke Registry and included 5,448 patients. Out of these only 48 patients (0.8%) presented with an isolated infratentorial bleeding.

The decision to include 14 isolated infratentorial / posterior fossa bleedings was chosen to be able to generate meaningful data for this bleeding type as well. It assumes that the sensitivity for the detection of these bleedings is 70%. In this case we would have a power of 80% to show that the lower bound of the corresponding 95%-CI interval of the sensitivity for these cases is above 50%. Please refer to section 11 for a detailed description of the calculation of the sample size.

3. Due to the nature of the intervention the enrolling physician and patients cannot be blinded to the intervention. Therefore, we opted for a blinded assessment of the endpoints to minimize bias. All readers in the first core-lab, who will assess the main endpoint will be blinded to all clinical data and will not participate in the enrollment of patients. In addition, a time-limit of 90 seconds per FDCT scan will be implemented to simulate real life conditions as in normal clinical practice the detection of intracranial hemorrhage often happens under considerable time pressure. The readers of the MDCT and FDCT scans will not be the same physicians and they will not share any information.

4. The non-inferiority approach was chosen to generate meaningful clinical evidence regarding the diagnostic abilities of non-contrast syngo DynaCT Sine Spin imaging. Non-inferiority trials for the evaluation of diagnostic modalities were done multiple times in different fields, highlighting the high value of these trials.^49, 50^ The non-inferior thresholds were derived from expert statements from highly experienced neuroradiologists/neurosurgeons.

## 6.2 Methods for minimizing bias

The components of the primary endpoint “occurrence of an intracranial hemorrhage” will be assessed by independent readers blinded to all clinical information. They will not be involved in the enrollment or treatment of any patient and will have no information on the distribution of patients (i.e. the number of patients with hemorrhage or without hemorrhage).

For this purpose, all scans will be reconstructed in a standardized manner first by the coordinating center to guarantee comparability between all scans. Fixed slice sizes and angels will be used for all scans. After reconstruction all scans will be pseudonymized and randomized. The order of the scans will differ for each rater. Furthermore, a time limit of 90 seconds will be used for the evaluation of the FDCT scans (only applicable to the binary decision hemorrhage yes/no) to ensure comparability to real-life conditions. No time-limit will be set for the MDCT scans as these are set as ground truth and therefore their diagnostic power will not be benchmarked.

### 6.2.1 Randomisation

Not applicable

### 6.2.2 Blinding procedures

Due to the nature of the procedure under investigation (diagnostic test) it is not possible, that study subjects, site investigators and care providers will be blinded. Furthermore, subjects are not randomized since all undergo the same procedures (cross-sectional design).

The main endpoint outcome parameter “occurrence of an intracranial hemorrhage” will be assessed by independent readers blinded to all clinical data. All scans will be reconstructed in a uniform matter and randomized prior to being assessed by the core-lab. Block randomization with variable block length will be used. The MDCT and FDCT scans will be read by different, independent readers. They will not have access to the results of other readers nor share any information about the clinical investigation with each other.

6.2.3 Other methods for minimising bias

1. Enrollment of patients will take place prior to the FDCT. Therefore, the enrolling physician could not know the appearance of the bleeding on the FDCT scan. This approach was chosen to rule out that enrolling physicians only include patients in which the FDCT scan is of high technical quality and the bleeding is well recognizable.

2. All readings will be documented on standardized forms.

## 6.3 Unblinding Procedures (Code break)

Not applicable

# 7. CLINICAL INVESTIGATION POPULATION

The clinical investigation will be conducted in Switzerland, Finland, France, Spain, and the USA. A preliminary list of all participating centers can be found in the appendix A.

## 7.1 Eligibility criteria

Subjects fulfilling all of the following inclusion criteria are eligible for the investigation:

- Informed Consent as documented by signature or fulfilling the criteria for emergency or deferral consent
- Patients with symptoms suggestive of ischemic stroke (NIHSS ≥ 7) or suggestive of haemorrhagic stroke with a cranial non-contrast MDCT and a feasible non-contrast syngo DynaCT Sine Spin within 4 hours
- Patient presenting within 24 hours of last seen well
- Patients presenting directly to the treating hospital (i.e. mothership patients) OR transfer patients with the indication for repeated imaging according to the standard operation procedures of the treating hospital*
- Age above 18 years
- Agreement of treating physician to perform non-contrast syngo DynaCT Sine Spin

The presence of any one of the following exclusion criteria will lead to the exclusion of the subject:

- Severe metal artifacts on initial MDCT imaging
- Planned invasive interventions between MDCT and FDCT scan
- Clinical deterioration between MDCT and FDCT scan (i.e. an increase of the NIHSS of more than 4 points)
- Evidence of an ongoing pregnancy prior to enrollment. A negative pregnancy test before enrollment is required for all women with child-bearing potential

*In case of transfer patients external MDCT images must be available at the PACS of the enrolling center prior to enrollment and the enrolling physician must ensure that the quality of these scans is sufficient for the assessment of the primary endpoint.

## 7.2 Recruitment and screening

Recruitment takes place in an emergency setting through usual care. No additional screening or examinations (except in women with child-bearing potential a pregnancy test) compared to normal clinical practice are necessary. The MDCT will be performed completely independently from the study.

All patients presenting to one of the participating sites with symptoms suggestive of acute stroke (i.e. NIHSS ≥7) or of an intracranial hemorrhage should be screened for this trial. In case of patients referred from other hospitals they are only considered for participation in this trial if they have an indication for repeated imaging according to local standards of care. For clarification repeated imaging can be done with FDCT, if external MDCT was of sufficient quality, is in the PACS of enrolling center, was within 4 hours of the anticipated FDCT, and the clinical presentation of the patient has not substantially changed (i.e., increase of NIHSS of more than 4 points). Patients will be recruited in the emergency or MDCT room. Primary responsibility for recruitment of patients will lie with the PI at each site.

Patients will not receive compensation for taking part in the trial.

## 7.3 Assignment to investigation groups

All included subjects will be assigned to the investigation group. There is no control group. All subjects undergo the same procedures.

## 7.4 Criteria for withdrawal / discontinuation of subjects

A trial patient or his/her next of kin/LAR can withdraw his/her consent to participate in the trial at any time and without any declaration of reason. In this case the data collected up to this time will be used for evaluation of the trial to not compromise the validity of the clinical trial or its results.^1^ After the final analysis the data will be anonymized. If a person who was included in a clinical trial in an emergency situation dies before it has been possible to obtain consent or refusal his/her data can only be used for analysis if there is a statement of wishes confirming the patients will to participate in clinical trials or if the consent is given by the next of kin of the patient,^1^ unless otherwise foreseen by national law. In case a person was included into the trial with the consent of an independent physician (due to his/her inability to give informed consent and the absence of a next of kin/LAR) and the patient refused to give informed consent after regaining the capacity to consent or his/her next of kin/LAR refuses to give informed consent (in case the patient did not regain the capacity to consent), all his/her data has to be erased.

# 8. CLINICAL INVESTIGATION INTERVENTION

## 8.1 Identity of the medical device under investigation

The following medical device will be used in the clinical investigation:

ARTIS icono biplane angiography system with syngo application software.

| Product/Trade Name: | Software version: | Model: | UDI-DI: |
| --- | --- | --- | --- |
| ARTIS icono biplane | VE21 or higher | 11327600 | 04056869063317 |
| syngo application software | VE21D or higher | 11327667 | 04056869124162 |

### 8.1.1 Experimental Intervention (medical device)

The experimental intervention is a non-contrast syngo DynaCT Sine Spin scan of the head. The scan duration is seven seconds and according to dose measurements the effective dose is up to 2.5 mSv.^3, 6^ It is a standard imaging protocol for visualization of the brain parenchyma and skull. It belongs to a bi-plane angiographic system the ARTIS Icono (Siemens Healthineers).

### 8.1.2 Control Intervention (standard/routine/comparator)

The control intervention is a non-contrast cranial MDCT scan. The system for the scan is left at the discretion of the site investigator. It is standard of care and done in all subjects irrespectively of their participation in the clinical investigation.

### 8.1.3 Labelling and Supply (re-supply)

This is a post-market device investigation with a CE-marked device. Therefore, labelling according to Art. 6.10 ISO14155 does not apply.

*See Appendix C- US Protocol Amendments

### 8.1.4 Storage Conditions

The medical device is permanently installed, and the required ambient conditions are considered for system installation. Key ambient conditions during system operation in the examination room, control- and equipment room include (see the ARTIS icono datasheet for further details):

• Temperature range: 15 °C – 30 °C

• Relative humidity: 20 – 75 % without condensation

• Max. temperature gradient: max. 5 °C/h

• Barometric pressure: 70 – 106 kPa

## 8.2 Discontinuation or modifications of the intervention

Because the clinical investigation intervention is a single intervention, discontinuation of the intervention is not an issue within this trial. Modifications of the intervention (i.e., changing the imaging protocol) is not permitted.

## 8.3 Compliance with clinical investigation intervention

Because the procedure under investigation is a single intervention there are no specific strategies to improve adherence to the protocol. Adherence will be monitored centrally, and deviation will be directly discussed with each trial site.

## 8.4 Data Collection and Follow-up for withdrawn subjects

As there are no follow-up visits planed there is no foreseeable scenario in which a patient might be lost to follow-up.

## 8.5 Clinical investigation specific preventive measures

All site PIs will be trained by the Sponsor-PI or the American chair in the conductance of the examined imaging protocol. Site Pis have to ensure that all personal performing the examination at their site is properly trained in executing it. Dose measurements were performed before in cooperation with Siemens Healthineers to ensure that the radiation of the used protocol does not exceed 2.5 mSv.^6^

## 8.6 Concomitant Interventions (treatments)

Not applicable.

## 8.7 Medical Device Accountability

Medical Device Accountability does not apply since the devices are permanently installed. The model and serial number of the systems will be recorded for each investigational site, also in case of a system exchange.

## 8.8 Return, Analysis or Destruction of the Medical Device

Since the medical device is CE-marked and permanently installed, the return of the system does not apply. In case of device deficiencies, including malfunction, usability issues, or inadequacy in the information supplied by the manufacturer including labelling, either on-site service or remote service is performed. In case of exchange of defective parts, these will be returned to the manufacturer for analysis as part of the complaint handling process.

# 9. CLINICAL INVESTIGATION ASSESSMENTS

## 9.1 Clinical investigation flow chart/ table of clinical investigation procedures and assessments


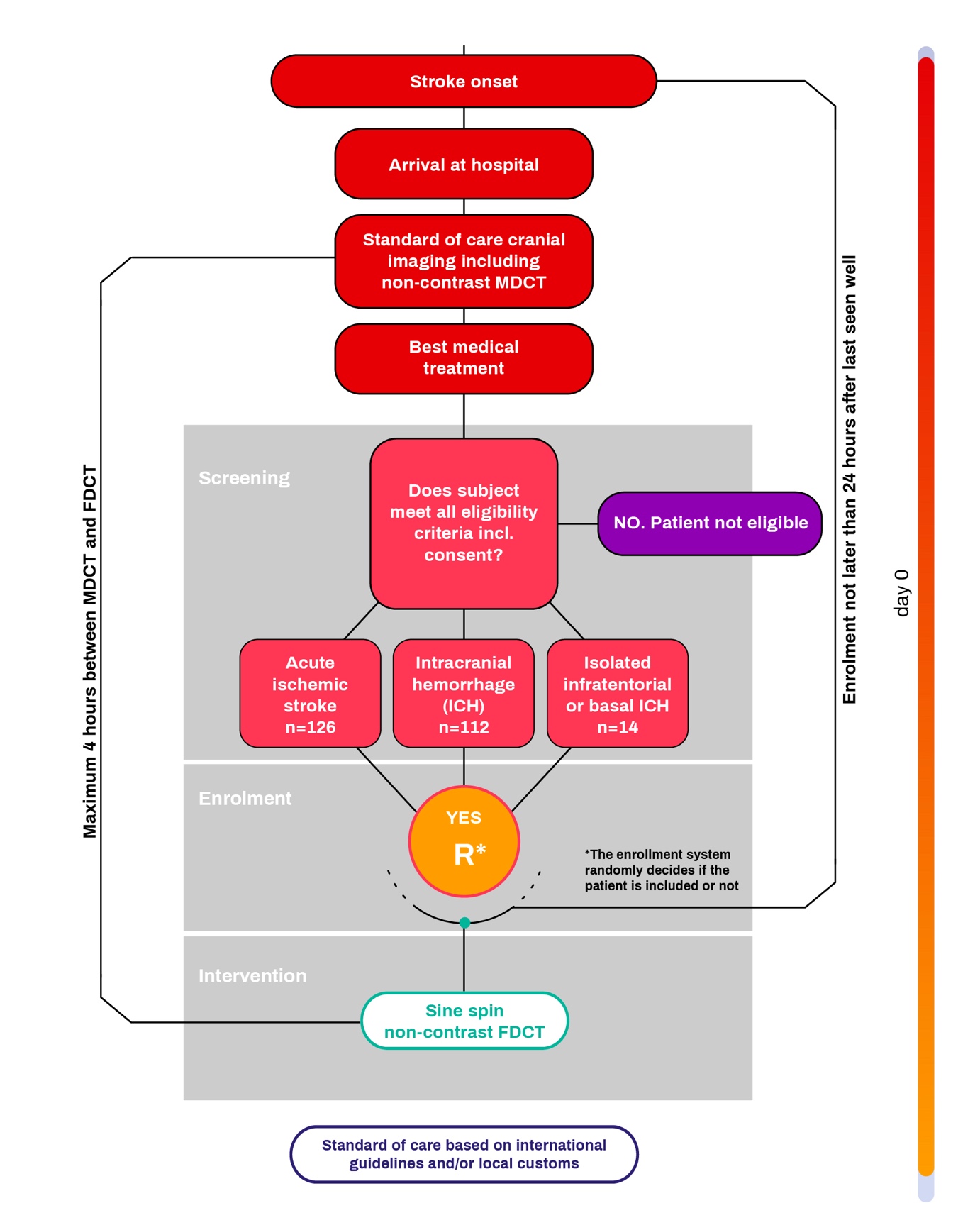


The Schedule of assessments to be performed at each study visit is listed in the table below

| **Schedule of assessments** | | | |
| --- | --- | --- | --- |
| **Visits** | | **0** | **1** |
|  |  | **Screening and enrollment** | **Intervention** |
| **Assessment** | **Time Window** | 0 | 0 – 4 hours after Non-contrast MDCT scan |
|  | **Method** | Clinical Visit | Intervention |
| Informed consent | | X^1^ |  |
| Patient demographics (i.e. age, gender, pre-existing conditions) | | X |  |
| National Institute of Health Stroke Scale | | X |  |
| Pregnancy test (only in women of childbearing potential) | | X |  |
| Patient logistics (i.e. time from onset to admission / imaging) | | X |  |
| Medical history (including medication) | | X |  |
| Non-contrast MDCT head scan^2^ | | X |  |
| Enrollment | | X |  |
| Non-contrast syngo DynaCT Sine Spin FDCT head scan | |  | X |
| AEs | | Only if procedure related up to 24 hours after procedure | |
| SAEs | | Only if procedure related up to 24 hours after procedure | |

1. Post-hoc consent if patient was not able to give consent at trial inclusion (according to national and applicable law)

2. For clarification the MDCT scan could have been done in an external hospital (in case of transfer patients) if the images of the MDCT are available at the PACS of the enrolling hospital and the quality was deemed to be sufficient by the enrolling physician and all other in- and exclusion criteria are met

AEs Adverse events, FDCT Flat-detector CT, MDCT Multi-detector CT, SAEs Serious adverse events

## 9.2 Assessments of outcomes

All outcomes will be assessed by a blinded and independent imaging core-lab. For this purpose, all scans will be anonymized, reconstructed in a standardized matter (i.e. fixed slice thickness 5 mm, distance 3 mm and angles - orbitomeatal) and randomized prior to evaluation.

The main core-lab will be provided by the Neurovascular Imaging Research Core at UCLA (Prof. David Liebeskind, University of California Los Angeles, Los Angeles, California, USA). The experience of the Neurovascular Imaging Research Core at UCLA as an imaging and angiography core lab is unrivaled, including more than 15,000 acute ischemic stroke cases treated with endovascular therapies in the last several years.

To ensure compliance with the Protocol and adequate acquisition technique of the FDCT and MDCT at the respective site the first five cases from each site will be deemed as lead-in cases. Cases can be excluded and deemed as Screen Failures if they show clear evidence of (a) operator failure (for example due to clear artifacts due to objects in the field of view, wrong positioning of head, wrong acquisition protocol, severe motion artifacts, missing reconstructions) or (b) technical failure due to missing calibration of the scanner. The ultimate decision to exclude cases is made by the Independent Core Lab (under the supervision of Prof David Liebeskind). In such a case it is the obligation of the Sponsor to perform retraining. Excluded cases will be replaced to ensure adequate power.

The imaging core-lab will use a standardized form to note all endpoints, in specific:

- Primary outcome:
  - Occurrence of an intracranial hemorrhage (yes/no)

The primary outcome will be assessed on both scans.

Only on the MDCT scans the following variables will be assessed as well:

- - Type of intracranial hemorrhage (SAH, intraparenchymal hemorrhage, intraventricular hemorrhage, subdural hemorrhage, epidural hemorrhage)
  - Is the intracranial hemorrhage isolated to the infratentorial region? (yes/no)

Only on the FDCT scans the following variables will be assessed as well:

- - Was contrast-media present at the evaluation of the scan? (yes/no)
  - If yes, did it enhance your capability to detect/exclude a hemorrhage? (yes/no)

The MDCT scans will be read by one clinical and one core-lab reader. If the reading of the core-lab reader and the clinical reader are consistent this will be defined as ground truth. In case of inconsistencies, a second core-lab (provided by Prof. Jan Gralla, Inselspital Bern) will be involved to define the ground truth (majority vote). Please refer to 6.1 for detailed information on the definition of ground truth.

All FDCT scans will be read by six readers. These readers will read all non-contrast syngo DynaCT Sine Spin scans independent from each other and share no information. They will be chosen in a way that it is secured that they do not work together or were trained together. None of the readers has seen the corresponding MDCT scans of the patient. In case most of the readers rate (at least 5 out of 6) an FDCT scan hemorrhage positive in a patient in whom the ground truth is hemorrhage negative, a second core-lab (provided by Prof. Jan Gralla, Inselspital Bern) will be involved. This core-lab will be unblinded to all clinical information and determine if the bleeding occurred between both scans. In this case the patient will be excluded from the analysis.

### 9.2.1 Assessment of primary outcome

The primary outcome “occurrence of intracranial hemorrhage” will be assessed by an independent blinded core-lab as detailed in 9.2

### 9.2.2 Assessment of secondary outcomes

See above under 9.2

### 9.2.3 Assessment of other outcomes of interest

There are no other outcomes of interest.

### 9.2.4 Assessment of safety outcomes

All AEs up to 24 hours after the procedure are investigated and documented in the source document. They are collected in the appropriate eCRF if they are deemed to be related to the procedure.

#### 9.2.4.1 Adverse events

All AEs, which are deemed to be procedure related by the local PI and occur during the conduct of the clinical investigation and/or within 24 hours of the intervention or till discharge (whatever happens earlier) of the subject are collected and documented on an ongoing basis. Due to the nature of the intervention, there are no foreseeable AEs

#### 9.2.4.2 Laboratory parameters

Not applicable

#### 9.2.4.3 Vital signs

Not applicable

### 9.2.5 Assessments in subjects who prematurely stop the clinical investigation

As there is only one visit, there is no foreseeable scenario in which a subject might prematurely stop the clinical investigation.

### 9.2.6 Follow-up of the subjects after the regular termination of the clinical investigation

No follow-up is planned.

## 9.3 Procedures at each visit

### 9.3.1 Visit 0 – Screening and enrollment

Stroke severity will be evaluated using the NIHSS in the emergency room. All subjects undergo a non-contrast MDCT scan (and CT angiography/perfusion if clinically indicated) immediately after admission. This MDCT scan will be done completely independently from the study as part of normal clinical routine. For clarification the MDCT scan could have been done at an external hospital (in case of transfer patients) provided the images were transferred to the PACS of the enrolling centre prior to enrolment and the enrolling physician has ensured adequate quality of the external MDCT scan.

Basic medical and surgical patient history will be obtained in all subjects according to local standard practice and in accordance with the AHA/ASA guidelines on stroke^44^ including medication, laboratory tests and vital signs. Women in childbearing age will be tested for pregnancy (study specific). A basic physical examination including NIHSS assessment will be performed. Intravenous thrombolysis should be administered immediately if the patient is deemed to be eligible under the international AHA/ASA guidelines^44^ and according to local standard practice. We highly recommend not to intubate the patient between the MDCT and FDCT scan.

Thereafter the subject will be screened for inclusion and exclusion criteria. All these subjects will be entered into the screening log (e.g., age, NIHSS, disapproved diagnosis). If the investigator comes to the decision that the subject qualifies for a trial participation, informed consent must be obtained immediately. If the investigator in agreement with the treating physician decides the subject is not able to give informed consent an independent physician has immediately to be organized. The independent physician must confirm or disapprove the trial participation for the specific subject.

*See Appendix C- US Protocol Amendments

### 9.3.2 Visit 1 - Intervention

Treatment will be continued for all subjects according to the international AHA/ASA guidelines^44^ and according to local standard practice.

After eligibility has been confirmed, the responsible investigator will access a 24-hour password protected, internet-based enrolment system to enrol the subject into the clinical investigation. The patient will be transported immediately to the angiography suite for subsequent treatment or further diagnostics. In the angiography suite non-contrast cranial syngo DynaCT Sine Spin imaging must be performed within four hours after the MDCT scan and before any other intervention or operation.

# 10. SAFETY

According to Swiss legislation there is no general obligation for the systematic documentation of adverse events (AEs) in medical device category A clinical investigations.^51^

**Adverse Events (AEs)** deemed to be related to the procedure under investigation (Adverse Device Effect) and all **Device deficiencies (DD)** are collected, fully investigated and documented in the source documents and appropriate case report forms (CRF) during the entire investigation period, i.e., from patient’s enrolment until the last CIP specific procedure, including a safety follow-up period of 24 hours. Serious adverse events which lead to death of the patient within 24 hours of enrolment will also be collected, fully investigated, and documented in the source documents and appropriate case report forms (CRF) independently of their causality.

## 10.1 Definition and Assessment of (Serious) Adverse Events and other safety related events

*See Appendix C- US Protocol Amendments

**Adverse Event (AE)** (Art. 2 Abs 57 MDR)

Any untoward medical occurrence, unintended disease or injury or any untoward clinical signs (including an abnormal laboratory finding) in subjects, users or other persons whether or not related to the MD.

**Serious Adverse Event (SAE)** (Art. 2 Abs 58 MDR)

Any adverse event that led to any of the following:

(a) death,

(b) serious deterioration in the health of the subject that resulted in any of the following:

(i) life-threatening illness or injury,

(ii) permanent impairment of a body structure or a body function,

(iii) hospitalisation or prolongation of patient hospitalisation,

(iv) medical or surgical intervention to prevent life-threatening illness or injury or permanent impairment to a body structure or a body function,

(v) chronic disease,

(c) foetal distress, foetal death or a congenital physical or mental impairment or birth defect.

Note: planned hospitalization for pre-existing condition, or a procedure required by the CIP, without a serious deterioration of the health status of the subject, is not considered an SAE. (ISO14155)

**Device deficiency** (Art. 2 Abs 59 MDR)

Inadequacy of a medical device related to its identity, quality, durability, reliability, safety or performance, of an investigational device, including malfunction, user errors and inadequate information supplied by the manufacturer.

**Malfunction** (ISO14155)

Failure of an investigational device to perform in accordance with its intended purpose when used in accordance with the instructions for use or the CIP.

**Device deficiency with Serious Adverse Device Effect (SADE) potential** (Art. 80 Abs 1 letter c MDR; ISO14155)

Any device deficiency that might have led to a serious adverse event if appropriate action had not been taken, intervention had not occurred, or circumstances had been less fortunate.

**Adverse Device Effect (ADE)** (ISO14155)

Adverse event possibly, probably or causally related to the use of an investigational device or procedures.

**Serious Adverse Device Effect (SADE)** (ISO14155)

Adverse device effect (ADE) that has resulted in any of the consequences characteristic of a serious adverse event.

**Unanticipated Serious Adverse Device Effect (USADE)** (ISO14155)

Serious adverse device effect (SADE) which by its nature, incidence, severity or outcome has not been identified in the current version of the risk analysis report.

**Causal Relationship of SAE** (MDCG 2020-10/1)

A causal relationship towards the medical device or the procedure of the investigation should be rated by the PI and the Sponsor as follows:

- **Not related:** The relationship to the device or procedures can be excluded.
- **Possible:** The relationship with the use of the investigational device is weak but cannot be ruled out completely. Alternative causes are also possible.
- **Probable:** The relationship with the use of the investigational device seems relevant and/or the event cannot reasonably be explained by another cause.
- **Causal relationship:** The serious event is associated with the investigational device or with procedures beyond reasonable doubt.

## 10.2 Adverse events categorization

The adverse events are categorized by the PI and the Sponsor using the following algorithm:

Does the AE meet the seriousness criteria?

- - No, it is not serious
    - - Is the relationship to the device or the procedure possible, probable or causal?
      - No: non-related AE
      - Yes: ADE
  - Yes, it is serious: SAE
    - - Is the relationship to the device or the procedure possible, probable or causal?
      - No: non-related SAE
      - Yes: SADE
  - Is it anticipated (within expected type, severity and frequency of the complications)?
    - - No: unanticipated SADE (USADE)
      - Yes: anticipated SADE (ASADE)

## 10.3 Documentation and reporting in Medical Device Category A clinical investigations

According to Swiss legislation there is no general obligation for the systematic documentation of adverse events (AEs) in medical device category A clinical investigations.^51^

*See Appendix C- US Protocol Amendments

**Adverse Events (AEs)** deemed to be related to the procedure under investigation (at least possible) and **Device deficiencies (DD)** are collected, fully investigated and documented in the source documents and appropriate case report form (CRF) during the entire investigation period, i.e. from patient’s enrolment until the last CIP specific procedure, including a safety follow-up period of 24 hours. Serious adverse events which lead to death of the patient within 24 hours of enrolment will also be collected, fully investigated, and documented in the source documents and appropriate case report forms (CRF) independently of their causality. These events will be coded according to the MedDRA system.

Documentation of SAEs by the PI includes diagnosis or symptoms, start and stop dates of event, event treatment, event resolution, assessment of seriousness and causal relationship to MD and/or investigation procedure (Art. 32 ClinO-MD, ISO14155).

Documentation of DDs by the PI includes description of event, start date, investigational device information, action taken with regard to the investigational device, and whether the DD led to an AE. The Sponsor shall review all DDs and determine and document in writing whether they could have led to a SAE (DD with SADE potential) (Art 32. ClinO-MD, ISO14155).

The Sponsor provides the CA and the CEC with the documentation at their request (Art. 32 ClinO-MD).

The information on AEs, DDs and SAEs will be collected through the eCRF. The follow-up period will be 24 hours after the intervention. Since the intervention has no invasive character (besides minor radiation), we do not expect the occurrence of any SAEs related to the medical device (or procedure/intervention) under investigation. For the occurrence of deterministic radiation damage (i.e. direct radiation damage) an radiation above 200 mSV must be applied. There is no scenario in which this could occur due to internal safety measures within the medical device under investigation. The internal safety measures are a) CAREwatch: Dose area product and the reference air kerma are shown on the screen, b) CAREmonitor: Display of the cumulated skin entrance dose of the currently radiated patient skin area and c) CAREguard: acoustic dose alert when the accumulated reference air kerma exceeds configurable thresholds.

### 10.3.1 Foreseeable adverse events and anticipated adverse device effects

There are no foreseeable adverse events or anticipated adverse device effects in this investigation.

### 10.3.2 Reporting of Safety related events Reporting to the Sponsor

All SAEs, device deficiencies and health hazards that require measures are reported to the Sponsor by the PI (or authorized designee) within 24 hours after becoming aware of the event. Device deficiencies are assessed regarding their potential to lead to an SAE.

Reporting to the Competent Ethics Committee:

The following events are to be reported to the CEC promptly (Art. 33 ClinO-MD):

1. any serious adverse event which has a causal relation with the MD (rating at least possible), comparator or procedure/test method or where a causal relation appears to be possible (SADE);
2. any device deficiency which, in the absence of appropriate measures or intervention or in less favourable circumstances, could have led to serious adverse events (DD with SADE potential);
3. any new information relating to an event already notified under points (1) and (2).

In order to ensure prompt notification, the Sponsor may initially submit an incomplete notification. If safety and health hazards that require measures must be taken immediately during the conduct of the investigation, the Sponsor notifies the CEC within 2 days of these measures and the circumstances which made them necessary (Art. 34 ClinO-MD).

**Periodic safety reporting (Art. 35 ClinO-MD):**

An Annual Safety Report (ASR) is submitted by the Sponsor to the CEC, yearly (Art. 35, 38 ClinO-MD). The ASR contains a list of all SADEs and DDs and a report on their degree of seriousness, causal relationship with the MD and procedure and on subjects’ safety. This includes all SADEs and DDs which have happened abroad.

### 10.3.3 Follow-up of (Serious) Adverse Events

SAEs if at least possibly related to the medical device under investigation, SADEs and SAIs will be followed until resolution or stabilization. Ongoing events must be documented at 14 days intervals until resolution. Subjects with ongoing events at trial termination will be further followed up until recovery or until stabilization after termination for another 14 days.

# 11. STATISTICAL METHODS

Detailed methodology for summaries and statistical analyses of the data collected in this study will be documented in a statistical analysis plan (SAP).

The SAP will be finalized before database closure and will be under version control at the Clinical Trial Unit, Department for Clinical Research, University Hospital Basel.

## 11.1 Hypothesis

Null hypothesis: Sensitivity and specificity of non-contrast contrast syngo DynaCT Sine Spin to detect the presence (sensitivity) and absence (specificity) of intracranial hemorrhages are inferior to MDCT. Inferiority is given when one of the lower bounds of the 95%-CI of sensitivity and specificity are equal or lower than 95%.

Alternative hypothesis: Sensitivity and specificity of non-contrast contrast syngo DynaCT Sine Spin to detect the presence (sensitivity) and absence (specificity) of intracranial hemorrhages are non-inferior to MDCT. Non-inferiority is given when both lower bounds of the 95%-CI of sensitivity and specificity are higher than 95%.

## 11.2 Determination of Sample Size

To calculate the sample size, hypothetical experiments were simulated. We estimate a sensitivity of 99% and a specificity of 98% of non-contrast syngo DynaCT Sine Spin imaging for the detection of intracranial hemorrhage as it was described for the predecessor generation in the literature.^48^ To account for variation, diagnostic performance of hypothetical readers was varied with rates of 98.5%, 99% and 99.5% for sensitivity and 97.5%, 98% and 98.5% for specificity. Rates were attributed randomly with equal chance.

Sensitivities and specificities of readers were generated from correlated binary random variables. To reproduce the correlated readings between readers, a high correlation between binary random variables was chosen. This assumption was made based on the literature and judgement of the principal investigators. We further simulated lower correlations which showed consistently that an approach using a high correlation is more conservative (i.e., leading to a higher sample size). Generated outcomes were vectors with 1 (true positive) and 0 (false negative) for sensitivity and 1 (true negative) and 0 (false positive) for specificity. The quotient of the occurrences of 1 and the number of cases with/or without a bleeding being sensitivity and specificity, respectively. In case sensitivity or specificity was 100%, a false negative (for sensitivity) and a false positive (for specificity) was introduced into the generated output. This enabled to still fit a model (see below).

To represent “difficult cases”, i.e. patients who present with an isolated infratentorial intracranial hemorrhage, we introduced a subpopulation of patients for whom the performance of raters was only 70% sensitivity. We chose n = 14 patients based on an additional sample size calculation. With this subsample, there is an 80% chance (power) that the lower bound of the 95%-CI of a sensitivity of 70% is higher than 50%. However, we assume that the subsample substantially overrepresents the incidence of difficult cases in the study population (see section 6.1). Therefore, we attributed a lower weight in the model to each difficult case.

It was tested whether the lower end of the 95%-CI did not fall below the prespecified margins. For sensitivity and for specificity this was 95%. The 95%-CI of sensitivity and specificity were calculated by fitting a generalized linear random-effects model with a logit link function. Random-effects variables were patient and reader, outcome variable was the randomly generated binary vector. To converge to model estimates, the algorithm of the “nloptwrap”-optimizer was applied.

From the model, the lower end of the 95%-CI of the fixed-effect intercept was extracted and transformed to percent. The 95%-CI was calculated by the method of Wald. If the lower ends of the 95%-CIs of sensitivity and specificity both did not fall below a pre-defined margin, the experiment was rated as “successful”. The percentage of “successful” experiment out of all experiments characterizes the study power. Each sample size, n_i=1,..,19_ = 80,…,260 was evaluated by sampling 999 times n_i_ individual samples with a range of readers from 1 to 7.

If the total weight of “difficult cases” is set at 0.02, the number of difficult cases at n= 14, the power at 90% the number of readers at 6, and the drop-out rate at 5%, a total of **252 patients** (238 patients without drop-outs) must be recruited. The patients must be pre-selected in a ratio of hemorrhage positive to hemorrhage negative of 1:1, i.e., 126 patients are presenting with and 126 patients without intracranial hemorrhage (out of which 14 present with an isolated infratentorial intracranial bleeding). The number of readers was set based on our model calculations since with a lower number of readers the sample size fluctuated substantially (over 100%). The needed sample size however stabilized at a range of 5 to 7 readers. With this number of readers, the sample size fluctuated only by 10% (with 7 readers being the lowest and with 5 the highest).

## 11.3 Readers

There will be 6 experienced readers (board certified neuro-interventionalists) who each rate all sine-spin non-contrast FDCT images on a two-point scale (presence or absence of intracranial hemorrhage). These readers will be selected in a way that they represent readers who typically rate such images in clinical routine setting. It will be secured that they do not work together or were trained together.

The sequence of the images to be read will random and differ for each reader. The readers will be blinded to all clinical data and to the clinical reading. To simulate clinical routine settings, a time limit (90 seconds) will be set for completion of the rating of the FDCT. All ratings will be noted on a standardized form. Ground truth will be deemed based on MDCT as described under 6.1. The reader of the MDCT will differ from the readers of the FDCT and they will not share any information. The number of hemorrhagic strokes and non-hemorrhagic strokes will not be disclosed to the readers.

## 11.4 Statistical criteria of termination of the investigation

None

## 11.5 Planned Analyses

### 11.5.1 Dataset to be analysed, analysis populations

For the analysis the full analysis set (FAS) will be used. The FAS contains all patients for which the six readers read all non-contrast syngo DynaCT Sine Spin images and rated them on a two-point scale (presence or absence of hemorrhage).

### 11.5.2 Primary Analysis

The primary analysis will be based on the FAS. As described for the sample size calculation, a generalized linear random-effects model with a logit link function will be fit. The explanatory variables ’reader’ and ’patients’ will be random effects. Difficult cases will be weighted with 2%. From the fixed-effect intercept of sensitivity and specificity, the 95%-CIs will be calculated applying the method of Wald. Sensitivity will be calculated based on the results of all core-lab readers of the non-contrast syngo DynaCT Sine Spin scans of all patients for which the ground truth was deemed to be hemorrhage positive. Specificity will be calculated based on the results of all core-lab readers of the non-contrast syngo DynaCT Sine Spin scans of all patients for which the ground truth was deemed to be hemorrhage negative. It will be tested whether both the lower bound of the 95%-CI of the sensitivity and specificity are higher than the margin of 95%.

All results will be reported in concordance with the STARD guidelines.^20^

### 11.5.3 Secondary Analysis

Fleiss’ kappa will be estimated to assess interrater reliability between 6 readers. Specificity, accuracy, positive predictive value, and negative predictive value will be calculated for FDCT.

For the subset of patients with an isolated infratentorial hemorrhage the sensitivity of FDCT for its detection including its 95%-CI will be reported separately.

11.5.4 Interim Analysis

No interim analysis is planned.

### 11.5.5 Deviation(s) from the original statistical plan

Changes to the statistical analysis plan will be under version control at the Clinical Trial Unit, Department for Clinical Research, University Hospital Basel.

## 11.6 Handling of missing data and drop-outs

The number and proportion of subjects eligible for and compliant with each follow-up examination will be presented. Careful planning and conduct of the study will minimise the occurrence of missing data as far as possible.

All analysis will be based on FAS. Missing data will be described but not imputed. For each patient that withdraws consent before the final outcome assessment, an additional patient is included.

# 12. QUALITY ASSURANCE AND CONTROL

## 12.1 Data handling and record keeping / archiving

### 12.1.1 Case Report Forms (CRF)

For each enrolled trial subject, an eCRF will be maintained. All data will be entered in coded way in the eCRF at the local site. eCRFs must be kept current to reflect subject status at each phase during the course of the trial and are part of the central acquisition by the CTU Basel. Coded identification for each patient will be as follow: [trial-ID]-[site-no]-consecutive number. Authorized to enter data into the eCRF are the local trial team staff according to the authorization list. Authorized persons will be identified by their usernames. The local PI is responsible for proper training and instruction of the trial personnel filling data into the eCRF.

### 12.1.2 Specification of source data and source documents

Source data must be available at the site to document the existence of the trial subjects. The following documents are source documents and can only be found at the local trial site:

- Informed consent form

- Patient screening, enrolment and identification log

- Patient records including reports from treating physician

- Documentation of the patients medical treatment

- Documented worksheets of scores (NIHSS)

- Radiological images from the FDCT and MDCT

- Radiology reports

- Any other relevant record to document AEs and SAEs

### 12.1.3 Archiving of essential clinical investigation documents

All trial data must be archived at the participating site for a minimum of 10 years or according to national requirements after trial termination or premature termination of the clinical trial. In addition, the electronic data will be kept for the mandatory period.

## 12.2 Data management

### 12.2.1 Data Management System

The eCRFs in this trial are implemented electronically using a dedicated electronic data capturing (EDC) system (secuTrial®). The EDC system is activated for the trial only after successfully passing a formal test procedure. All data entered in the eCRFs are stored on a Linux server in a dedicated Oracle database. Responsibility for hosting the EDC system and the database lies with the University Hospital Basel.

### 12.2.2 Data security, access and back-up

The server hosting the EDC system and the database is kept in a locked server-room. Only the system administrators have direct access to the server and back-up tapes. A role concept with personal passwords (site investigator, statistician, monitor, administrator etc.) regulates permission for each user to use the system and database as he/she requires.

All data entered into the eCRFs are transferred to the database using Secure Sockets Layer (SSL) encryption. Each data point has attributes attached to it identifying the user who entered it with the exact time and date. Retrospective alterations of data in the database are recorded in an audit table. Time, table, data field, original value and altered value, and the user are recorded (audit trail).

A multi-level back-up system is implemented.

### 12.2.3 Analysis and archiving

For the final analyses, data files will be extracted from the database after final database closure into statistical packages to be analysed. The status of the database at this time will be recorded in special archive tables. The trial database with all archive tables will be securely stored by the CTU Basel and the University Hospital Basel for at least 10 years. The Sponsor-Investigators will also keep the Trial Master File and interim/final reports for at least 10 years.

### 12.2.4 Electronic and central data validation

Data is checked by the EDC system for completeness and plausibility. Furthermore, selected data points are cross-checked for plausibility with previously entered data for that subject. In addition, central data reviews will be performed on a regular basis to ensure completeness of the data collected and accuracy of the primary outcome data.

Before database lock the local PI will validate the collected data with his signature.

## 12.3 Monitoring

All source data must be accessible for auditing and monitoring. Monitors and auditors will maintain patient confidentiality. All questions from the monitors will be answered during the monitoring.

A risk-based monitoring approach will be implemented for this trial. The risks of the trial will be assessed by the Sponsor-Investigators. A multi-level monitoring plan will be defined based on these risks. It will describe the regular standardized and manual checks of the entered data in the trial database, the remote monitoring activities and the on-site monitoring visits. The Sponsor-Investigators will delegate the monitoring tasks to qualified institutions upon individual agreement.

## 12.4 Audits and Inspections

Authorities have the right to perform inspections, and the University Hospital Basel has the right to perform on-site auditing during working hours upon reasonable prior notice. The auditor/inspector must have access to all medical records, the investigator's trial related files and correspondence, and the informed consent documentation that is relevant for this clinical trial.

PIs will allow the persons responsible for the audit or the inspection to have access to the source data/documents and PIs will answer any questions arising. All involved parties will keep the patient data strictly confidential.

## 12.5 Confidentiality, Data Protection

The information contained in this protocol is copyright protected by the University Hospital Basel. This information is given for the needs of the trial and must not be disclosed to persons outside of the trial community without prior written consent of the University Hospital Basel.

Trial-related data of the patient will be provided in a coded way. The names of the patients will not be disclosed to persons outside of a participating site. A unique patient identification number will be attributed to each patient registered into the trial (for more details see section 12.1.1). Identification of patients must be guaranteed at each site using the patient screening, enrolment and identification list. In order to avoid identification errors, patient’s identification number and the year of birth have to be provided on the eCRF. Patient confidentiality will be maintained according to applicable legislation.

Direct access to source documents will be permitted for purposes of monitoring, audits and inspections. The monitoring institution (i.e., CTU Basel or Semmes Murphey Foundation or another institution authorized by the Sponsor-Investigator) and the local authorities will have access to all information necessary for such tasks during and after the study.

Regarding the data specified in Appendix B (“Specified Data”), University Hospital Basel (the Sponsor) grants Siemens Healthineers a non-exclusive, unrestricted, perpetual, fully paid-up, transferable and sublicensable right to use and/or have used the Specified Data, in any manner whatsoever (in particular to reproduce, distribute, including the right to lease and the right to make available to the public). This grant of rights includes the right of Siemens Healthineers to use the Specified Data in unmodified or modified form, alone or in combination with other data, in all known and unknown ways for the purpose of applying for regulatory approval (i.e. device clearances, registration certification and/or responding to requests of authorities), education, marketing and/or sales of devices and/or services. According to University Hospital Basel`s evaluation the Specified Data contain (amongst others) Personal Data. Siemens Healthineers and its Affiliates will process and disclose Personal Data to third parties only for the following purposes: applying for regulatory approval (i.e. device clearances, registration certification and/or responding to requests of authorities) and/or education. Selected images generated from the specified data can be used for marketing and/or sales of devices and/or services after prior written approval of the University Hospital Basel.

## 12.6 Storage of related health data

All health-related data will be stored at the local recruiting site at a secure location. Coded data of all scans (Non contrast MDCT and non contrast syngo DynaCT Sine Spin) will be stored on a secure server hosted by the Department of Research, Clinic of Nuclear Medicine and Radiology, University Hospital Basel and made available over a HIPAA-secure connection to the ICL at the Neurovascular Imaging Research Core at UCLA (Los Angeles, California, USA). For some participants the image will also be made available over a secure connection to the second ICL at the Inselspital Bern. The images will only be stored temporarily at the ICLs for the time of the conductance of the Core-Lab.

# 13. PUBLICATION AND DISSEMINATION POLICY

Results of the SPINNERS clinical investigation will be reported according to the applicable STARD statement (<https://www.equator-network.org/reporting-guidelines/stard/>).^20^ The results of the SPINNERS trial will be published in high-ranked open-access international scientific journals. Furthermore, trial results will be distributed by national and international clinical networks and through national and international organizations and societies (e.g. European Stroke Organisation, American Heart Association).

# 14. FUNDING AND SUPPORT

The trial is funded by a grant from Siemens Healthineers.

# 15. INSURANCE

In Switzerland, the University Hospital Basel will indemnify patients for any damage they may suffer due to participation in this trial. For this purpose, the University Hospital Basel has taken out a special insurance for clinical trials with Helvetia Schweizerische Versicherungsgesellschaft:

Police Number 4.001.396.623

For all other countries individual patient insurance will be arranged according to national and local requirements. A copy of the insurance certificate will be issued in each investigator site file and the trial master file.

# 16. REFERENCES

1. Humanforschungsgesetz, hfg bundesgesetz über die forschung am menschen (bundesgesetz über die forschung am menschen, hfg) vom 30. September 2011/ loi fédérale relative à la recherche sur l’être humain (loi relative à la recherche sur l’être humain, lrh) du 30 septembre 2011 / legge federale concernente la ricerca sull'essere umano (legge sulla ricerca umana, lrum) del 30 settembre 2011. .

2. Ordinance on clinical trials in human research, <https://www.Fedlex.Admin.Ch/eli/cc/2013/643/en> accessed on the 04/29/2021.

3. Brehm A, Stamm G, Lüpke M, Riedel C, Stieltjes B, Psychogios MN. Effective dose to patient measurements for flat-detector computed tomography protocols in acute stroke care. *Eur Radiol*. 2020;30:5082-5088

4. Verordnung über klinische versuche mit medizinprodukten (klinv-mep) vom 1. Juli 2020 / ordonnance sur les essais cliniques de dispositifs médicaux (oclin-dim) du 1er juillet 2020 /. Ordinanza sulle sperimentazioni cliniche con dispositivi medici (osrum-dmed) del 1 luglio 2020.

5. Humanforschungsgesetz, hfg, <https://www.Fedlex.Admin.Ch/eli/cc/2013/617/de> accessed on the 04/29/2021.

6. Petroulia VD, Kaesmacher J, Piechowiak EI, Dobrocky T, Pilgram-Pastor SM, Gralla J, et al. Evaluation of sine spin flat detector ct imaging compared with multidetector ct. *Journal of NeuroInterventional Surgery*. 2022:neurintsurg-2021-018312

7. Román LS, Menon BK, Blasco J, Hernández-Pérez M, Dávalos A, Majoie CBLM, et al. Imaging features and safety and efficacy of endovascular stroke treatment: A meta-analysis of individual patient-level data. *The Lancet Neurology*. 2018;17:895-904

8. Goyal M, Menon BK, van Zwam WH, Dippel DWJ, Mitchell PJ, Demchuk AM, et al. Endovascular thrombectomy after large-vessel ischaemic stroke: A meta-analysis of individual patient data from five randomised trials. *The Lancet*. 2016;387:1723-1731

9. Saver JL, Goyal M, van der Lugt A, Menon BK, Majoie CBLM, Dippel DW, et al. Time to treatment with endovascular thrombectomy and outcomes from ischemic stroke: A meta-analysis. *JAMA*. 2016;316:1279-1289

10. Psychogios MN, Bähr M, Liman J, Knauth M. One stop management in acute stroke: First mothership patient transported directly to the angiography suite. *Clin Neuroradiol*. 2017;27:389-391

11. Psychogios MN, Maier IL, Tsogkas I, Hesse AC, Brehm A, Behme D, et al. One-stop management of 230 consecutive acute stroke patients: Report of procedural times and clinical outcome. *J Clin Med*. 2019;8

12. Requena M, Olivé M, García-Tornel Á, Rodríguez-Villatoro N, Deck M, Juega J, et al. Time matters: Adjusted analysis of the influence of direct transfer to angiography-suite protocol in functional outcome. *Stroke*. 2020;51:1766-1771

13. Jadhav AP, Kenmuir CL, Aghaebrahim A, Limaye K, Wechsler LR, Hammer MD, et al. Interfacility transfer directly to the neuroangiography suite in acute ischemic stroke patients undergoing thrombectomy. *Stroke*. 2017;48:1884-1889

14. Requena M, Olivé-Gadea M, Muchada M, Hernández D, Rubiera M, Boned S, et al. Direct to angiography suite without stopping for computed tomography imaging for patients with acute stroke: A randomized clinical trial. *JAMA Neurol*. 2021

15. Heldner MR, Hsieh K, Broeg-Morvay A, Mordasini P, Bühlmann M, Jung S, et al. Clinical prediction of large vessel occlusion in anterior circulation stroke: Mission impossible? *J Neurol*. 2016;263:1633-1640

16. Duvekot MHC, Venema E, Rozeman AD, Moudrous W, Vermeij FH, Biekart M, et al. Comparison of eight prehospital stroke scales to detect intracranial large-vessel occlusion in suspected stroke (presto): A prospective observational study. *The Lancet Neurology*. 2021;20:213-221

17. Eckert M, Gölitz P, Lücking H, Struffert T, Knossalla F, Doerfler A. Optimized flat-detector ct in stroke imaging: Ready for first-line use? *Cerebrovascular Diseases*. 2017;43:9-16

18. Leyhe JR, Tsogkas I, Hesse AC, Behme D, Schregel K, Papageorgiou I, et al. Latest generation of flat detector ct as a peri-interventional diagnostic tool: A comparative study with multidetector ct. *J Neurointerv Surg*. 2017;9:1253-1257

19. Hemphill JC, 3rd, Greenberg SM, Anderson CS, Becker K, Bendok BR, Cushman M, et al. Guidelines for the management of spontaneous intracerebral hemorrhage: A guideline for healthcare professionals from the american heart association/american stroke association. *Stroke*. 2015;46:2032-2060

20. Bossuyt PM, Reitsma JB, Bruns DE, Gatsonis CA, Glasziou PP, Irwig L, et al. Stard 2015: An updated list of essential items for reporting diagnostic accuracy studies. *Radiology*. 2015;277:826-832

21. Emanuel EJ, Wendler D, Grady C. What makes clinical research ethical? *JAMA*. 2000;283:2701-2711

22. Khatri P, Abruzzo T, Yeatts SD, Nichols C, Broderick JP, Tomsick TA. Good clinical outcome after ischemic stroke with successful revascularization is time-dependent. *Neurology*. 2009;73:1066-1072

23. Khatri P, Yeatts SD, Mazighi M, Broderick JP, Liebeskind DS, Demchuk AM, et al. Time to angiographic reperfusion and clinical outcome after acute ischaemic stroke: An analysis of data from the interventional management of stroke (ims iii) phase 3 trial. *Lancet Neurol*. 2014;13:567-574

24. Jahan R, Saver JL, Schwamm LH, Fonarow GC, Liang L, Matsouaka RA, et al. Association between time to treatment with endovascular reperfusion therapy and outcomes in patients with acute ischemic stroke treated in clinical practice. *JAMA*. 2019;322:252-263

25. Sarraj A, Goyal N, Chen M, Grotta JC, Blackburn S, Requena M, et al. Direct to angiography vs repeated imaging approaches in transferred patients undergoing endovascular thrombectomy. *JAMA Neurol*. 2021;78:916-926

26. Lozano R, Naghavi M, Foreman K, Lim S, Shibuya K, Aboyans V, et al. Global and regional mortality from 235 causes of death for 20 age groups in 1990 and 2010: A systematic analysis for the global burden of disease study 2010. *The Lancet*. 2012;380:2095-2128

27. Feigin VL, Forouzanfar MH, Krishnamurthi R, Mensah GA, Connor M, Bennett DA, et al. Global and regional burden of stroke during 1990&#x2013;2010: Findings from the global burden of disease study 2010. *The Lancet*. 2014;383:245-255

28. Murray CJL, Vos T, Lozano R, Naghavi M, Flaxman AD, Michaud C, et al. Disability-adjusted life years (dalys) for 291 diseases and injuries in 21 regions, 1990&#x2013;2010: A systematic analysis for the global burden of disease study 2010. *The Lancet*. 2012;380:2197-2223

29. Kunz WG, Hunink MG, Almekhlafi MA, Menon BK, Saver JL, Dippel DWJ, et al. Public health and cost consequences of time delays to thrombectomy for acute ischemic stroke. *Neurology*. 2020;95:e2465

30. Declaration of helsinki, version october 2013 (<http://www.Wma.Net/en/30publications/10policies/b3/index.Html> )

31. Medical device regulation (eu) 2017/745 of 5 april 2017 (mdr) (<https://eur-lex.Europa.Eu/legal-content/en/txt/?Uri=celex%3a32017r0745>).

32. Ich harmonised guideline integrated addendum to ich e6(r1): Guideline for good clinical practice ich e6(r2) ich consensus guideline. <Https://ichgcp.Net/> accessed on the 04/29/2021.

33. Singh D, Wasan H, Reeta KH. Preclinical stroke research and translational failure: A bird's eye view on preventable variables. *Cell Mol Neurobiol*. 2021

34. Hill MD, Goyal M, Menon BK, Nogueira RG, McTaggart RA, Demchuk AM, et al. Efficacy and safety of nerinetide for the treatment of acute ischaemic stroke (escape-na1): A multicentre, double-blind, randomised controlled trial. *The Lancet*. 2020;395:878-887

35. Saver JL. Time is brain--quantified. *Stroke*. 2006;37:263-266

36. Jovin TG, Albers GW, Liebeskind DS. Stroke treatment academic industry roundtable: The next generation of endovascular trials. *Stroke*. 2016;47:2656-2665

37. Schregel K, Behme D, Tsogkas I, Knauth M, Maier I, Karch A, et al. Effects of workflow optimization in endovascularly treated stroke patients – a pre-post effectiveness study. *PLOS ONE*. 2016;11:e0169192

38. Brehm A, Tsogkas I, Ospel JM, Appenzeller-Herzog C, Aoki J, Kimura K, et al. Direct to angiography suite approaches for the triage of suspected acute stroke patients: A systematic review and meta-analysis. *Therapeutic Advances in Neurological Disorders*. 2022;15:17562864221078177

39. Struffert T, Richter G, Engelhorn T, Doelken M, Goelitz P, Kalender WA, et al. Visualisation of intracerebral haemorrhage with flat-detector ct compared to multislice ct: Results in 44 cases. *Eur Radiol*. 2009;19:619-625

40. Psychogios MN, Buhk JH, Schramm P, Xyda A, Mohr A, Knauth M. Feasibility of angiographic ct in peri-interventional diagnostic imaging: A comparative study with multidetector ct. *American Journal of Neuroradiology*. 2010;31:1226

41. Rao NM, Levine SR, Gornbein JA, Saver JL. Defining clinically relevant cerebral hemorrhage after thrombolytic therapy for stroke: Analysis of the national institute of neurological disorders and stroke tissue-type plasminogen activator trials. *Stroke*. 2014;45:2728-2733

42. Eckert M, Gölitz P, Lücking H, Struffert T, Knossalla F, Doerfler A. Optimized flat-detector ct in stroke imaging: Ready for first-line use? *Cerebrovasc Dis*. 2017;43:9-16

43. Payabvash S, Khan AA, Qureshi MH, Saeed O, Suri MF, Qureshi AI. Detection of intraparenchymal hemorrhage after endovascular therapy in patients with acute ischemic stroke using immediate postprocedural flat-panel computed tomography scan. *J Neuroimaging*. 2016;26:213-218

44. Powers WJ, Rabinstein AA, Ackerson T, Adeoye OM, Bambakidis NC, Becker K, et al. Guidelines for the early management of patients with acute ischemic stroke: 2019 update to the 2018 guidelines for the early management of acute ischemic stroke: A guideline for healthcare professionals from the american heart association/american stroke association. *Stroke*. 2019;50:e344-e418

45. Cortnum S, Sørensen P, Jørgensen J. Determining the sensitivity of computed tomography scanning in early detection of subarachnoid hemorrhage. *Neurosurgery*. 2010;66:900-902; discussion 903

46. Blok KM, Rinkel GJ, Majoie CB, Hendrikse J, Braaksma M, Tijssen CC, et al. Ct within 6 hours of headache onset to rule out subarachnoid hemorrhage in nonacademic hospitals. *Neurology*. 2015;84:1927-1932

47. Perry JJ, Stiell IG, Sivilotti ML, Bullard MJ, Emond M, Symington C, et al. Sensitivity of computed tomography performed within six hours of onset of headache for diagnosis of subarachnoid haemorrhage: Prospective cohort study. *Bmj*. 2011;343:d4277

48. Leyhe JR, Tsogkas I, Hesse AC, Behme D, Schregel K, Papageorgiou I, et al. Latest generation of flat detector ct as a peri-interventional diagnostic tool: A comparative study with multidetector ct. *Journal of neurointerventional surgery*. 2017;9:1253-1257

49. Madsen JR, Boyle TP, Neuman MI, Park EH, Tamber MS, Hickey RW, et al. Diagnostic accuracy of non-invasive thermal evaluation of ventriculoperitoneal shunt flow in shunt malfunction: A prospective, multi-site, operator-blinded study. *Neurosurgery*. 2020;87:939-948

50. Snelling PJ, Keijzers G, Byrnes J, Bade D, George S, Moore M, et al. Bedside ultrasound conducted in kids with distal upper limb fractures in the emergency department (buckled): A protocol for an open-label non-inferiority diagnostic randomised controlled trial. *Trials*. 2021;22:282

51. Verordnung über klinische versuche mit medizinprodukten (klinv-mep) from the 01. July 2020; downloaded from <https://www.Fedlex.Admin.Ch/eli/cc/2020/553/de> (accessed on the 31.05.2021).

# 17. Appendices

A. Preliminary Side List

B. Specified Data

C. US Amendments to Protocol

D. eCRF (separate document)

E. Monitoring plan (separate document)

F. Informed consent forms (separate document)

G. Additional information on data protection only applicable to France (separate document)

**Appendix A: Preliminary Site List**

Sites are mentioned on basis of preferred candidates. These are provisional lists, and may be updated through a selection process that USB will decide on.

SWITZERLAND

- Universitätsspital Basel
- Inselspital, Universitätsspital Bern
- Luzerner Kantosspital

FRANCE

- CHRU Tours

FINLAND

- HUS Helsinki
- Turku

SPAIN

- Val Hebron Barcelona Hospital

USA

- Semmes Murphy, Memphis, TN
- Advocate Lutheran General, Chicago, IL
- University of Texas Southwestern Medical Center, Dallas, TX
- Ascension Wisconsin Hospital, Greenfield, WI
- Mount Sinai Hospital, New York, NY
- University of Pennsylvania, Philadelphia, PA
- NorthShore University HealthSystem, Evanston, IL
- University of Kentucky, Lexington, KY
- Swedish Medical Center, Englewood, CO
- New York University, New York, NY
- MGH Institute of Health Professions, Boston, MA
- Washington University, St Louis, MO
- The University of Iowa, Iowa City, IA

**Appendix B: Specified Data**

Definition for the European sites:

- Deidentified (i.e., all information in DICOM tags which would allow for identification of patients have to be removed or replaced with a code) DICOM data: ideally, all diagnostic neuroimaging scans acquired in the study population (with or without intracranial hemorrhage) with both imaging modalities used for comparative purposes, i.e. *syngo DynaCT Sine Spin* and conventional MDCT (multidetector computed tomography). All DICOM data must be provided as follows
- FDCT: 3D-reconstructed volume data (as per CoreLab specification)
- MDCT: reconstructed slice images (as per CoreLab specification)
- All information needed to allow for comparison of neuroimaging scans acquired by both beforementioned modalities such as imaging protocols, time between scans, administration of contrast medium (if any) incl. time of administration etc.
- Per intracranial hemorrhage observed: clinical description and classification including (but not limited to) sub-type, location / anatomical region
- Detailed description of lesions mimicking bleeding (if any) in *syngo DynaCT Sine Spin* images of patients without intracranial hemorrhage (a posteriori documentation)
- Neuroimaging scans/data used for CoreLab quality check on possible impact of contrast medium administration (intra-individual) between the neuroimaging scans with both beforementioned imaging modalities

wherein for data from **US Sites** it shall be deemed sufficient if Specified Data meets the following criteria:

- Deidentified (i.e., all information in DICOM tags which would allow for identification of patients have to be removed or replaced with a code) DICOM data: ideally, all diagnostic neuroimaging scans acquired in the study population (with or without intracranial hemorrhage) with both imaging modalities used for comparative purposes, i.e. *syngo DynaCT Sine Spin* and conventional MDCT (multidetector computed tomography). All DICOM data must be provided as follows
- FDCT: 3D-reconstructed volume data (as per CoreLab specification)
- MDCT scans will be provided according to standard of practice at each US site; at the minimum 1-5 mm axial slices per each subject, coronal and sagittal images if available (as per CoreLab specification)
- All information needed to allow for comparison of neuroimaging scans acquired by both beforementioned modalities such as imaging protocols, time between scans, administration of contrast medium (if any) incl. time of administration etc.
- Per intracranial hemorrhage observed: clinical description and classification including (but not limited to) sub-type, location / anatomical region
- Detailed description of lesions mimicking bleeding (if any) in *syngo DynaCT Sine Spin* images of patients without intracranial hemorrhage (a posteriori documentation)
- Neuroimaging scans/data used for CoreLab quality check on possible impact of contrast medium administration (intra-individual) between the neuroimaging scans with both beforementioned imaging modalities

**Appendix C: US Amendments to Protocol**

Section 2.2- US medical device classification Class 2. Minimal risk to subject (up to 2.5 mSv according to internal measurements and recent publications)^6^

Section 2.3- WCG Clinical- Connexus will serve as the central IRB

Section 2.7- In the US an LAR will be requested if present OR a deferral of consent will be requested for post-hoc consent from subjects who are unable to give consent pre investigation.

Section 2.8- Subject identifiers at US sites will not leave each individual site. A reference record will be kept on site for subject verification and eligibility. However, this record will not leave the site and will only be accessible by site staff, monitoring and Sponsor through encryption.

Section 2.10- All amendments to this study will be sent to Connexus-WCG for approval.

Section- 2.11 Any deviation that harms a subject or places them at risk (referred to as Promptly Reportable Information) will be reported to Connexus- WCG in accordance with their policies. All other deviations will be reported at the annual Continuing Review.

Section 3.2

| Product/Trade Name: | Software version: | Model: | FDA 510(k) Number: |
| --- | --- | --- | --- |
| ARTIS icono biplane | VE21 or higher | 11327600 | K201156 |
| syngo application software | VE21D or higher | 11327667 | K190780 |

Section 3.3- Devices have been approved for use by FDA

Section 8.1.3- This is a post-market device investigation

Section 9.3.1 In the US in case the patient is not able to give consent due to his or her acute illness and no LAR is present deferral of consent for will be used and consent will be obtained post-hoc.

Section 10.1- Code of Federal Regulations Title 21 Section 312.32 IND Safety Reporting will be used in the US for reference in Adverse Events. Code of Regulations Title 21 Part 803 will be used for Device deficiency and Malfunction reporting

Section 10.3- Adverse Events will be reported in accordance with FDA guidelines previously outlined in previous amended sections. Yearly data safety reports will be submitted by the DSMB.
