# Supplementary material for: ProSPective evaluation of the dIagnostic accuracy of siNe spiN non-contrast flatdEtectoR CT (FDCT) for the detection of intracranial hemorrhage in stroke patients - Protocol of a non-inferiority comparison to multi detector CT
Source: PLoS One. 2025 Aug 28;20(8):e0330608. doi: 10.1371/journal.pone.0330608 (PMC12393753; doi:10.1371/journal.pone.0330608)
Supplement: Attachment — Submitted filename: Rebuttal_Letter_170725.pdf [file pone.0330608.s008.pdf]

University Hospital Basel

Radiology and Nuclear Medicine
Diagnostic and Interventional Neuroradiology

Marios Psychogios, MD
Section Chief

University Hospital Basel
Petersgraben 4
CH-4031 Basel

email Marios.Psychogios@usb.ch

personal assistant Yasmine Wick
phone +41 61 328 63 70
fax +41 61 265 43 54
email yasmin.wick@usb.ch
website www.unispital-basel.ch

Basel, 17 July 2025

Dear Dr. Meckel,

First, we want to express our gratitude for your and the reviewer's evaluation of the manuscript. Important points were raised, which improve the quality of the manuscript.
We have updated the trial status on page 10 line 261 – 262 to reflect the status.
Please find attached a point-by-point reply to the points raised:

Journal Requirements:

Please review your reference list to ensure that it is complete and correct. If you have cited papers that have been retracted, please include the rationale for doing so in the manuscript text, or remove these references and replace them with relevant current references. Any changes to the reference list should be mentioned in the rebuttal letter that accompanies your revised manuscript. If you need to cite a retracted article, indicate the article's retracted status in the References list and also include a citation and full reference for the retraction notice.

We have reviewed the reference list and ensured its completeness. One reference was incorrect numbered; we have corrected this. In specific in this sentence reference 6 was included but 7 was the correct reference:
"We estimate a sensitivity of 99% and a specificity of 98% of non-contrast Sine Spin FDCT imaging for the detection of intracranial hemorrhage as described for the predecessor generation in the literature. [7]"

Reviewer #1:

The queries have been addressed adequately. Please check for typos. For example , on the revision, page 10, line 251 'Durin' should be 'During'.

Chairman E. Merkle, MD **Vice-Chairman** D. T. Boll, MD **Senior attendings** J. Bremerich, MD I S. Dellas, MD I T. Heye, MD I A. Hirschmann, MD I M. Psychogios, MD I D. Wild, MD PhD I Ch. Zech, MD **Chief Technologist** B. Schaedeli

Thank you very much for highlighting this typo. We worked with a native speaker through the manuscript to improve grammar and correct all typos.

Reviewer #2:

Thanks for addressing the previous comments and questions. I also have some comments for the current version:

1. In ischemic and hemorrhagic stroke, the authors may provide the definition such as ICD-10 or some other source for disease diagnosis.

Thank you for your suggestion. In our study, the diagnosis of ischemic and hemorrhagic stroke is made in the acute setting, primarily in the emergency room. At this stage, clinical evaluation (such as an acute neurological deficit assessed using NIHSS or GCS) combined with imaging findings on multidetector CT (MDCT) forms the basis for diagnosis. Since these assessments occur before formal coding and discharge documentation, referencing an ICD-10 code is not appropriate in this context.

Furthermore, our inclusion criteria focus on patients who present via a pre-hospital stroke alert and are received as suspected acute stroke cases. This approach ensures timely triage and intervention, which is critical in the acute management of stroke.

We have also clarified this in the protocol paper and added under “Population” (Page 6 line 149 – 153) the following sentence: *“The diagnosis of an ischemic or hemorrhagic stroke is made in the acute setting, primarily in the emergency room, prior to patient enrollment. At this stage, clinical evaluation—such as the assessment of acute NIHSS or the Glasgow Coma Scale (GCS) — combined with imaging findings from the MDCT forms the basis for diagnosis.”*

Thank you for considering the manuscript for publication,

Marios Psychogios and Adam S Arthur
